# Simultaneous modeling of reaction times and brain dynamics in a spatial cueing task

**DOI:** 10.1002/hbm.25758

**Published:** 2021-12-24

**Authors:** Simon R. Steinkamp, Gereon R. Fink, Simone Vossel, Ralph Weidner

**Affiliations:** ^1^ Cognitive Neuroscience Institute of Neuroscience & Medicine (INM‐3), Research Centre Juelich Juelich Germany; ^2^ Department of Neurology Faculty of Medicine and University Hospital Cologne, University of Cologne Cologne Germany; ^3^ Department of Psychology, Faculty of Human Sciences University of Cologne Cologne Germany

**Keywords:** behavioral, dynamic causal modeling, effective connectivity, fMRI, simultaneous modeling, spatial attention

## Abstract

Understanding how brain activity translates into behavior is a grand challenge in neuroscientific research. Simultaneous computational modeling of both measures offers to address this question. The extension of the dynamic causal modeling (DCM) framework for blood oxygenation level‐dependent (BOLD) responses to behavior (bDCM) constitutes such a modeling approach. However, only very few studies have employed and evaluated bDCM, and its application has been restricted to binary behavioral responses, limiting more general statements about its validity. This study used bDCM to model reaction times in a spatial attention task, which involved two separate runs with either horizontal or vertical stimulus configurations. We recorded fMRI data and reaction times (*n*= 26) and compared bDCM with classical DCM and a behavioral Rescorla–Wagner model using Bayesian model selection and goodness of fit statistics. Results indicate that bDCM performed equally well as classical DCM when modeling BOLD responses and as good as the Rescorla–Wagner model when modeling reaction times. Although our data revealed practical limitations of the current bDCM approach that warrant further investigation, we conclude that bDCM constitutes a promising method for investigating the link between brain activity and behavior.

## Graphical abstract

To understand how brain and behavioral dynamics are linked, it is essential to model both simultaneously. An approach that allows this to be done is behavioral dynamic causal modeling (bDCM), which we further extend to continuous behavioral readouts. In the current study, we explore its applications and limitations and demonstrate that bDCM performs similarly or better than single modality models.

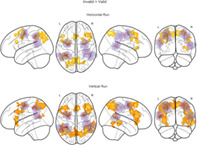

## 1 INTRODUCTION

Computational modeling can deepen our understanding of how the brain processes information and produces overt behavior. In psychology, computational modeling has a long history of describing and explaining behavioral concepts. For example, reinforcement learning algorithms have been used to explain classical conditioning (Rescorla & Wagner, 1972), drift‐diffusion models have been used to model reaction times in decision‐making tasks (Ratcliff, 1978), and race models of reaction times have been used as theoretical formulations of visual–spatial attention (Bundesen, 1990). Similarly, different computational modeling approaches have been employed in neuroscience and neuroimaging. For example, generative graphical models of brain connectivity describing blood oxygenation level‐dependent (BOLD) amplitudes in response to experimental inputs can be estimated using dynamic causal modeling (DCM) (Friston et al., 2017; Friston, Harrison, & Penny, 2003), and multivariate temporal response functions have been used to model ongoing sensory stimulation, like speech, in electrophysiological recordings (Crosse, Di Liberto, Bednar, & Lalor, 2016).

Although computational models are very prominent in the two fields, behavioral and neural responses are mostly treated separately (Turner, Forstmann, Love, Palmeri, & van Maanen, 2017). However, a combined modeling framework could provide deeper insights into the neural processes and the emergence of behavior. Different approaches have been proposed here: one possibility is to correlate the parameters of neural and behavioral models to describe how the different measures are related across different participants (Vossel, Weidner, Moos, & Fink, 2016). Alternatively, in model‐based fMRI, the behavioral computational model's outputs (or hidden states) are used as a factor in a classical GLM analysis. One such factor could be a participant's perceived cue validity in a probabilistic spatial cueing task, recovered from reaction times (e.g., Dombert, Kuhns, Mengotti, Fink, & Vossel, 2016). Leveraging the theory‐driven outputs of cognitive models allowed to determine more specific brain activation patterns of cognitive processes than by using nonspecific measures such as reaction times (Turner et al., 2017). A third option is a joint modeling approach (Turner et al., 2017). Here, an overarching set of parameters is used to describe both brain activity and behavior. An example is a study by Nunez (2015), where the drift‐diffusion model parameters were constrained with task‐based brain activity, incorporating the covariation between reaction times and neural activity on a trial‐by‐trial basis.

Although these approaches are tremendously useful, none of them employs an integrative model describing the generation of brain activity and behavior, which would allow us to directly investigate the hidden processes behind the two measurements. Rigoux and Daunizeau (2015) provided such a framework, where an additional output function extends DCM to describe behavioral responses (behavioral DCM, bDCM). This simultaneous modeling has high descriptive power and allows thorough diagnostics of the model. For example, by disabling specific nodes in the network (i.e., artificial lesions), conclusions can be drawn about the contribution or necessity of different brain regions to the emergence of behavioral patterns. So far—to our knowledge—bDCM has been applied to a larger dataset in one study only, which modeled binary choices in an economic decision‐making task (Shaw et al., 2019).

The current study shows that bDCM can be extended to continuous measures (i.e., reaction times). Furthermore, we provide a direct comparison between bDCM and classical DCM, and between bDCM and an adjusted version of the Rescorla–Wagner model (Rescorla & Wagner, 1972; Vossel et al., 2014). We employ Bayesian model comparison based on the free energy of competing models and classical metrics of accuracy (mean absolute error and *R* ^2^‐score).

As a testing ground, we modeled the effects of attentional reorientation along the horizontal and vertical meridians in a spatial cueing paradigm, where participants had to report the orientation of a pre‐cued Gabor patch. In trials in which invalid cues indicated an incorrect location of the target Gabor patch (20% of the trials), participants had to reorient their attention to the opposite location (Posner, 1980). This paradigm has been found to elicit reliable reaction time differences between invalid and valid trials, both on the individual and the group level (Hedge, Powell, & Sumner, 2017). Additionally, it has been shown that the internal representation of cue validity can be modeled using the Rescorla–Wagner model as a generative model of reaction times (Mengotti, Dombert, Fink, & Vossel, 2017; Vossel, Mathys, et al., 2014).

Besides the reliable behavioral effects, the cortical networks involved in this task have been characterized by multiple studies. We have previously analyzed the present dataset using classical DCM (Steinkamp, Vossel, Fink, & Weidner, 2020), which has also been used in similar cueing paradigms (c.f., Vossel, Weidner, Driver, Friston, & Fink, 2012). Moreover, studies in patients with stroke‐induced lesions have revealed brain regions critically involved in spatial cueing tasks (Corbetta & Shulman, 2011; Malherbe et al., 2018; Posner, Walker, Friedrich, & Rafal, 1984). It is well established that the orientation of visual–spatial attention is mediated by a dorsal fronto‐parietal attention network consisting of the intraparietal sulci (IPS) and the frontal eye fields (FEF). This network interacts with a ventral fronto‐parietal attention network of ventral frontal cortex and the temporoparietal junction (TPJ) when a sudden reorientation of attention is necessary (Corbetta, Kincade, Lewis, Snyder, & Sapir, 2005; Corbetta & Shulman, 2011). In patients with spatial neglect, damage to ventral parietal regions such as TPJ causes a deficit in reorienting to contralesional targets. Moreover, it leads to dysfunctions in structurally intact dorsal regions such as the IPS (Corbetta et al., 2005), and direct lesions to the IPS have also been associated with impaired reorienting (Gillebert et al., 2011).

As IPS, FEF, and TPJ may differentially contribute to the behavioral outcome (RT), we used Bayesian model comparison to identify which regions convey information about the behavioral dynamics after accounting for the complexity of the network model.

## 2 METHODS

### 2.1 Participants

Data were collected from 29 participants (15 female, 21–39 years old, *M* = 25, *SD* = 3) with normal or corrected‐to‐normal vision [all right‐handed, Edinburgh handedness Inventory (Oldfield, 1971), *M* = 0.86, *SD* = 0.14], who provided written informed consent to participate in the study. Participants had to be older than 18 and younger than 40 years old and had to be right‐handed. Participants with neurological or psychiatric disorders were excluded from the study. Due to the fMRI protocol, we also excluded participants with metal implants and tattoos. One participant had to be excluded subsequently because of noncompliance. Another participant was excluded due to excessive head movement (predefined criteria translation >3 mm, rotation >3°). Furthermore, we could not extract the time series for the left‐TPJ volume of interes (VOI) in one participant. Therefore, the final sample included 26 participants. The ethics board of the German Psychological Association had approved the study. Volunteers were paid 15€ per hour for their participation. The dataset has been used in a previous study (see Steinkamp et al., 2020).

### 2.2 Task

**FIGURE 1:**
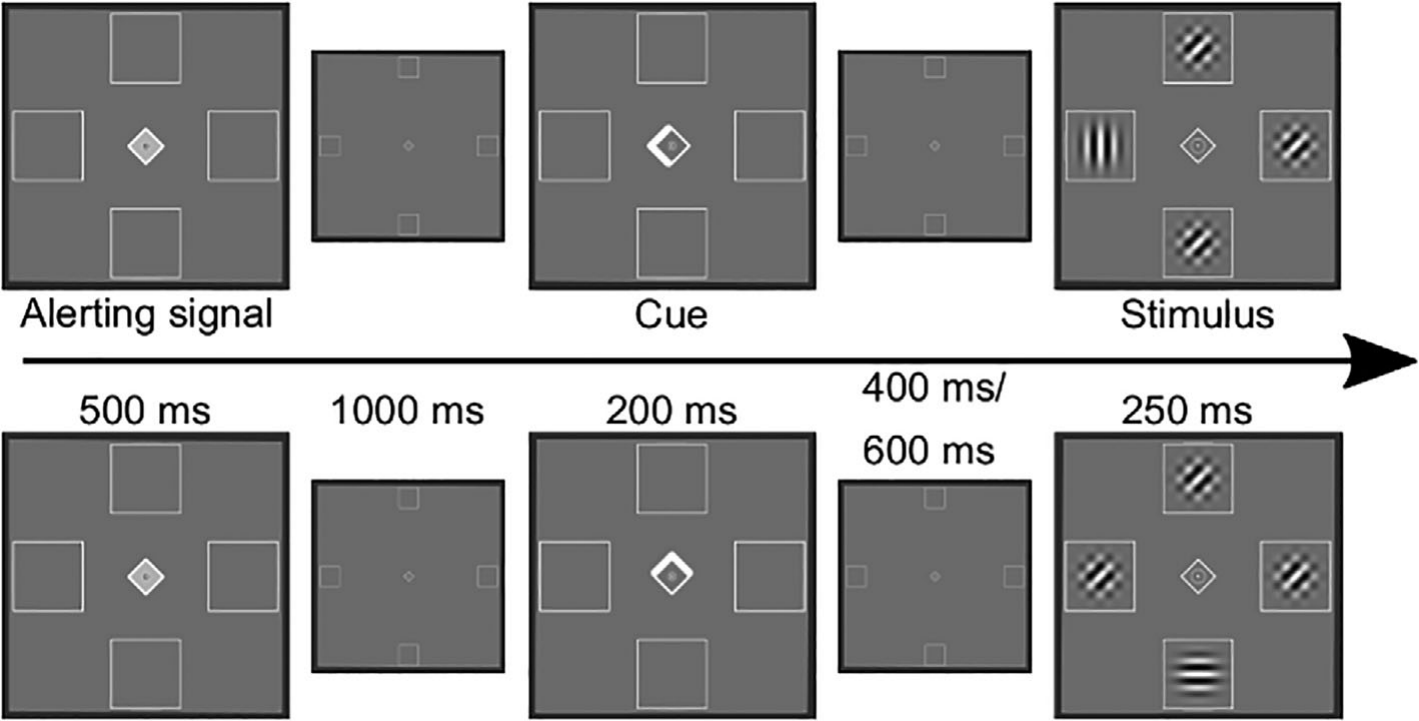
Illustration of the spatial cueing paradigm. In the upper row, a valid trial of the horizontal run is shown. The lower row depicts an example of an invalid trial in the vertical run. Reused from Steinkamp et al. (2020), licensed under a Creative Commons Attribution 4.0 International License (https://creativecommons.org/licenses/by/4.0/)

Participants performed a spatial cueing task while lying in a 3 T Trio (Siemens, Erlangen) MRI scanner. Stimuli were displayed on a screen behind the scanner bore, which could be seen via a mirror (mirror to display distance: 245 cm) mounted on a 32‐channel head coil. The participants' task was to report the orientation (horizontal/vertical) of a target Gabor patch (size 1° visual angle) by button presses of either the left or the right index finger while continuously fixating a diamond in the center of the screen (0.5° visual angle). A brightening of the central diamond (500 ms) indicated the beginning of a trial and was followed by a spatial cue after 1,000 ms (brightening of one of the diamond's edges for 200 ms) that indicated the location of the following target stimulus with 80% probability. Participants were explicitly informed about the percentage of cue validity. The possible target locations were indicated by empty boxes (1° width) located to the fixation diamond's left, right, top, and bottom (4° visual angle). After 400 or 600 ms, the target stimulus appeared for 250 ms at the cued location or in the box opposite to it. Distractor stimuli (constructed from two overlapping Gabor patches that were rotated by −45° and 45°, respectively) appeared simultaneously in the remaining three locations. Participants performed two runs of the spatial cueing paradigm. In one run, targets and cues occurred along the vertical axis, in another along the horizontal axis (see Figure 1).

Each run consisted of five blocks of 40 trials (32 valid, 8 invalid). All possible combinations of target location, target orientation, and interstimulus interval were presented in random order within each block. The time between the trials was drawn from the set of 2.0, 2.7, 3.2, 3.9, or 4.5 s with equal probability. Between the blocks, there was a break of 10–13 s.

Run order (vertical or horizontal first) and the response mapping (left index finger for vertical orientations/right index finger for horizontal orientations or vice versa) were counterbalanced across participants. Before the experiment, participants performed a rapid detection task to train the mapping of stimulus–response associations. Here, targets appeared rapidly in the middle of the screen, and participants had to press the corresponding button as fast as possible. Immediate feedback and a running score of their accuracy were given. Additionally, there were 20 practice trials with feedback before each run of the main experiment.

Stimulus presentation and response collection were controlled using PsychoPy (version 1.85.3, Peirce, 2007, 2008; Peirce et al., 2019).

### 2.3 Behavioral analysis

The mean reaction times were calculated for each participant, cueing condition, and target location. Before calculating the mean reaction times, we preprocessed the data for each participant separately. First, incorrect, missed, and outlier trials were removed. Outliers were defined as trials with reaction times below 0.2 s or greater than the 75th percentile + 3 × interquartile range (IQR). The higher threshold for outlier exclusion was chosen to retain as many trials as possible in the analysis (removed trials, including errors, in the horizontal run: invalid *M* = 2.54, *SD* = 2.63; valid *M* = 6.62, *SD* = 5.91; in the vertical run: invalid *M* = 3.12, *SD* = 1.8; valid *M* = 6.0, *SD* = 3.94).

For the analysis of the “validity effect” (i.e., the slowing of reaction times in invalid as compared with valid trials), the data were pooled across the two runs (horizontal/vertical). The mean reaction times of the 2 × 4 (cueing × target location) factorial design were then analyzed in a repeated‐measures ANOVA. The analysis was conducted in Python 3.7 using pingouin (version 0.3.3; Vallat, 2018).

### 2.4 fMRI analyses

For each participant and each run, we collected 557 T2*‐weighted images using an echo‐planar imaging (EPI) sequence [time of repetition (TR) 2.2 s; echo time (TE) 30 ms; flip angle 90°]. Each recorded volume consisted of 36 transverse slices with a slice thickness of 3 mm and a field of view of 200 mm. The voxel size was 3.1 × 3.1 × 3.3 mm. The first five images were discarded to account for T1 equilibrium artifacts. Next to functional images, we also obtained an anatomical T1‐weighted image for each participant, which was used in the preprocessing.

We preprocessed the fMRI data using fmriprep (version 1.1.1; Esteban et al., 2019), a robust and standardized pipeline, which applies slice‐time correction, realignment, and normalization to MNI space. A detailed preprocessing report can be created automatically (see http://fmriprep.readthedocs.io/en/1.1.1/workflows.html) and is included in the Supporting Information.

Data was further spatially smoothed using an 8 × 8 × 8 mm FWHM Gaussian kernel. This step was done in Matlab 2018b (The MathWorks, Inc., Natick, Massachusetts), using SPM12 (version 7.771; Friston, 2007).

### 2.5 fMRI—GLM

A classical GLM analysis was performed to identify activation peaks during attentional orientation and reorientation, later used to extract BOLD time‐series data for the DCM analysis. The GLM analysis was conducted using SPM12. First‐level models were created with four regressors of interest for each run, representing invalidly cued targets on the left (iL) and on the right (iR), validly cued targets on the left (vL) and the right (vR) for the horizontal run, and invalidly and validly cued targets in the lower (iD, vD) and the upper (iU, vU) part of the screen in the vertical run.

To account for other physiological noise in the BOLD signal, we added the three rotation and three translation estimates of the rigid body transform, the average white matter signal, and the average cerebral spinal fluid (CSF) signal as nuisance regressors. We further included the squared time‐series of the eight regressors, the time‐shifted time‐series (*t* − 1), as well as the square of the shifted time‐series, resulting in a total of 32 nuisance regressors (Friston, Williams, Howard, Frackowiak, & Turner, 1996). We also applied a high pass filter at 128 s. For each run, four first‐level contrasts were calculated: T‐contrasts of valid and invalid trials versus baseline, an F‐contrast of target onset versus baseline, which was used in the VOI analysis, and a differential contrast of invalid trials greater than valid trials. The latter contrast isolates brain regions involved in attentional reorientation.

At the group (second)‐level, we investigated the differential contrast of invalid > valid trials using two planned one sample permutation *t*‐tests against 0 using SnPM 13 (Nichols & Holmes, 2002), with default settings, 10,000 permutations, and no additional variance smoothing, using the initial set of 27 participants. The cluster forming threshold was estimated during the processes with a predefined voxel‐level cutoff of *p* < .001.

### 2.6 Modeling analysis

In the following, we will describe the modeling approaches used in our analysis, followed by a description of our model assessments and further analyses.

#### 2.6.1 Rescorla–Wagner model

We employed a variant of the Rescorla–Wagner model used previously (Mengotti et al., 2017). While this study was interested in the α parameter (the learning rate that describes how quickly participants adjust their internal assessment of the cue‐validity), we applied this modeling approach to simulate reaction times in a trial‐by‐trial fashion. For parameter estimation, we defined new functions for the variational Bayesian analysis (VBA) toolbox (clone from master, in January 2020, Daunizeau, Adam, & Rigoux, 2014).

We used the following reinforcement learning formula as the evolution function, describing the hidden process governing the generation of reaction times:

$$v_{t}=v_{\left(t-1\right)}+\alpha *\delta_{t}$$

where $\delta_{t}=u_{t}-v_{\left(t-1\right)}$ describes the prediction error at trial t. The external input $u_{t}\in \left[0,1\right]$ describes whether the cue at time t was either valid (0) or invalid (1), α is the learning rate, and $\;v_{t}$ is the participant's perceived cue invalidity (i.e., the probability, that the cue will be invalid) after observation of trial t.

The observation function (i.e., the mapping from perceived cue invalidity to reaction times) was defined as:

$$g_{t}=u_{t}*\left(\zeta_{i}+\zeta_{2}*v_{t-1}\right)+\left(1-u_{t}\right)*\left[\zeta_{v}+\zeta_{2}*\left(1-v_{t-1}\right)\right]$$

According to this formulation, the perceived cue invalidity of the previous trial governs the responses, with different bias parameters for valid and invalid trials and a general scaling parameter of the predictions.

To keep the behavioral dynamics as close as possible to the observed data, we set the reaction time of missing and outlier trials to 0 but ignored these trials during model inversion. The mean and standard deviation over participants of the posterior estimates can be found in the Section S6 in Supporting Information.

Table 1 depicts the Gaussian priors used in our estimation.

**TABLE 1:** Overview of parameters and prior values of the Rescorla–Wagner model

Parameters	μ	σ	
α	0.5	0.5	To ensure 0<α≤1, α was logit and inverse logit transformed during parameter updating
$\zeta_{v}$	0	1	
$\zeta_{i}$	0	1	
$\zeta_{2}$	0	1	
$v_{0}$	0.5	1	Initial state of v

#### 2.6.2 Behavioral DCM

In the following, we will provide a short overview of key concepts of DCM. For a full derivation and detailed description of DCM (see, Friston et al., 2003; Rigoux & Daunizeau, 2015; Stephan et al., 2008). DCM is a full Bayesian approach to create a generative model of brain dynamics and infer effective connectivity between selected brain regions. In principle, DCM describes how experimental variations (described by the input u) drive the neural activity (x, the hidden states) in brain regions of interest in a dynamical system. The evolution function $\left[\dot{x}=f\left(x,u\right)\right]$ describes the temporal dynamics of the hidden states ($\dot{x}$) and how they are influenced by external inputs (u). In DCM for fMRI, the evolution function f is typically described as:

$$f\left(x,u,\theta \right)\hat{=}\mathrm{Ax}+\sum_{j}u_{j}B^{j}x+\mathrm{Cu}$$

j corresponds to the number of inputs. The neural evolution parameters in θ correspond to the entries in A (fixed connectivity between brain regions), $B^{j}$ (modulation of connection strength by input j), and C (direct effects of inputs). Hemodynamic states *z* (dependent on the neural states *x*) are then gated through an observation function:

$$y=g\left(z,\phi \right)+\epsilon$$

This function captures BOLD signal variations based on the hemodynamic states (z) and the hidden neural activity (x), with hemodynamic parameters ϕ. This mapping allows to observe and infer the hidden neural dynamics via the BOLD signal.

BDCM augments the described formulation of DCM by adding new hidden states (r) for observed responses. The dynamic of r is defined by $r_{t+1}=h\left(x,u\right)-\alpha r_{t}$, with $h\left(x,u,\psi \right)$ as an additional evolution function, and $g_{r}\left(r\right)+\epsilon_{r}$ as the observation function to map the hidden neural dynamics to behavioral responses. The evolution function h of the new “behavioral” state follows the same rationale as the function f in the DCM formulation:

$$h\left(x,u,\psi \right)\hat{=}\;A_{r}x+\sum_{j}u_{j}B_{r}^{j}x+C_{r}u.$$

Here the parameter vector ψ describes the linear ($A_{r}$) components of the behavioral state and the direct ($C_{r}$) and modulatory ($B_{r}^{j}$) influences of experimental manipulations. $A_{r}$ is an analogy of the weight vector in a regression model. In the original article, the neural states were mapped to binary behavioral observations (button press absent or present) via a sigmoidal function:

$$s\left(r\right)=\frac{1}{1+e^{-100*\left(\rho +r\right)}}$$

Here, ρ is an unknown bias term, and r is the response or decision state. In our study, we slightly adjusted the sigmoid mapping by changing the scale on which it operates. As we are not expecting reaction times slower than 3 s, we used this as an upper bound:

$$s\left(r\right)=\frac{3}{1+e^{-100*\left(\rho +r\right)}}$$

##### Regions

As in our previous study (Steinkamp et al., 2020), we included bilateral IPS and FEF in our DCM model, which correspond to the central nodes of the dorsal fronto‐parietal attention network (Vossel, Geng, & Fink, 2014). Additionally, as part of the ventral attention network, we included the TPJ bilaterally. As additional inclusions (e.g., the inferior/middle frontal gyrus) would have increased model complexity and computational resources (and time), we did not include other brain regions, which may also play a role in attention reorienting.

Based on our assumptions about the dorsal and ventral attention network's interplay, we created three automatic meta‐analyses using NeuroSynth (https://www.neurosynth.org/; Yarkoni, Poldrack, Nichols, van Essen, & Wager, 2011) to define the seed coordinates for the subsequent VOI analysis (see Table 2). Our regions of interest were bilateral IPS (search term: “intraparietal sulcus”), bilateral FEF (search term: “frontal eye”), and bilateral TPJ (search term: “tpj”). We downloaded the corresponding association maps (associations, *p* < .01 FDR corrected) and identified the seed location as the peak voxel in the cluster of interest, using the Anatomy toolbox (v2, Eickhoff et al., 2005). In all three maps, the two largest clusters encompassed our regions of interest in either the left or right hemispheres.

**TABLE 2:** Regions and search‐terms for automated NeuroSynth meta‐analyses

Region	NeuroSynth (accessed 10.10.19)	*Z*‐statistic	*X*	*Y*	*Z*
IPS—Left	“Intraparietal sulcus”	14.6	−30	−50	42
IPS—Right	“Intraparietal sulcus”	13.5	40	−38	44
FEF—Left	“Frontal eye”	13.9	−30	−4	52
FEF—Right	“Frontal eye”	14.6	32	−6	52
TPJ—Left	“Tpj”	8.56	−60	−54	20
TPJ—Right	“Tpj”	11.4	58	−50	14

We used the participant level *t*‐maps (thresholded at *p* < .1 uncorrected) in each run to search for individual local maxima in a 12 mm sphere around the seed coordinates. The first principal component of BOLD time‐courses in a 9 mm VOI around the participant's maximum was extracted and adjusted based on the F‐contrast defined in the first‐level analysis. Task‐related activity for the IPS and FEF VOIs was defined by the contrast of valid trials against baseline and for TPJ by the contrast of invalid trials against the baseline.

##### Preprocessing

We preprocessed the BOLD signal by detrending each VOI signal (*spm_detrend*) and scaling the BOLD amplitude across VOIs to a maximum value of 4 (see *spm_dcm_estimate*). Behavioral data were extracted from the event data, and as in the previous analyses, error trials, trials with missed responses, and RTs fulfilling the outlier criterion (RT < 0.2 s and RT > 3 × IQR + UQ) were excluded.

BOLD data were resampled from a TR of 2.2 s to a sampling rate of 1.1 s (by interspersing “NaN” values). The behavioral observations were set to occur at the corresponding target onset, which was also downsampled to a resolution of 1.1 s. No resampling of BOLD data was performed for the classical DCM analysis. As the Rescorla–Wagner model represents trial‐by‐trial dynamics, the corresponding preprocessed reaction times were used, excluding error, missed, and outlier trials.

For our modeling, we assumed homogenous HRF dynamics across the six regions, fixing the initial states of the model to 0, and estimating the shape of the observation noise hyper‐prior distributions. For this, we assumed that we would be able to explain 10–90% of the variance in both the BOLD and the reaction time data. The prior distributions over the other parameters were set to the defaults of the VBA toolbox. We used the same hyperpriors for the explained variance of the BOLD signal in the classical DCM analysis and the Rescorla–Wagner model's behavioral responses.

To define the inputs into the DCM models, we created separate SPM‐design matrices that were only used to define the input streams. Stream one (u_1_) was defined as the driving input to all six regions, containing an impulse every time a target stimulus appeared (irrespective of the cueing condition or target location). The second stream (u_2_) was used purely for the modulatory effects, containing an impulse only in invalidly cued targets. The input streams were extracted from the SPM design matrix and were centered before entering the model inversion (*spm_detrend*). As mentioned above, the Rescorla–Wagner model is modeling trial‐by‐trial variations (rather than continuous time), so the input to this model was a vector consisting of ones and zeros, indicating whether the current trial is invalid or valid.

##### Model definition and comparison

**FIGURE 2:**
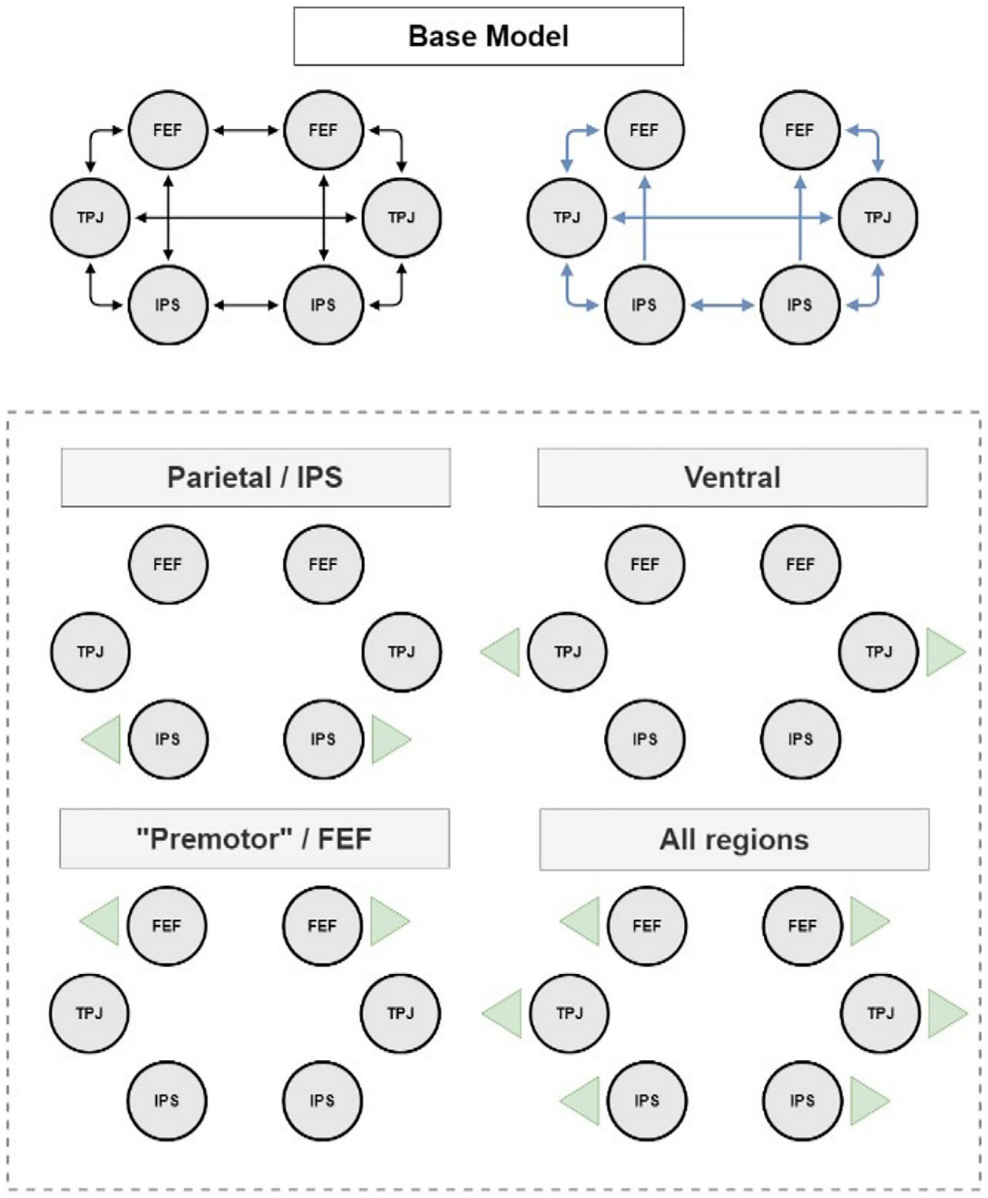
Top row, basic structure of the DCM and bDCM models. Regions were fully interconnected in each hemisphere, and homologous regions were connected. All regions received driving input. We assumed that all regions' connections were modulated by invalid trials, except for feedback and interhemispheric connections from FEF. In the lower part, we conducted Bayesian model selection to select the most plausible output region(s), indicated by green arrows

Before bDCM was compared against models of single modalities (classical DCM and Rescorla–Wagner model, respectively), we conducted Bayesian model selection to identify the most plausible configuration of output connections (i.e., the region in which neural activity is linked to the behavioral output). We used IPS, FEF, and TPJ as our brain regions of interest as described above. The fixed connectivity structures of our model (i.e., the A‐matrix) had complete connections in each hemisphere and between homologous regions (Figure 2). As we did not include visual areas in our modeling approach, all six regions received driving input (C‐matrix). For bidirectional intrahemispheric and interhemispheric modulatory connections (B‐matrix), we considered the IPS and TPJ. Connections in both hemispheres to the FEF were unidirectional, assuming that there were no feedback modulations from FEF to the other brain regions. We then investigated how neural dynamics in the included brain regions are related to behavioral dynamics. More specifically, we tested the following alternative hypotheses regarding bDCM's $A_{r}$ matrix:

- Neural activity in IPS drives behavior. IPS is a major hub region in the dorsal attention network. It is thought to initiate top‐down modulation of visual areas when attention is oriented in space. Moreover, it is often co‐activated together with ventral frontoparietal regions during attentional reorienting in invalid trials, and isolated IPS lesions in stroke patients can lead to reorienting impairments (Gillebert et al., 2011; Vossel, Geng, & Fink, 2014).
- Neural activity in FEF drives behavior. FEF is also part of the dorsal attention system and is crucially involved in covert and overt attentional orienting (Corbetta, Kincade, & Shulman, 2002; Rizzolatti, Riggio, Dascola, & Umiltá, 1987).
- Neural activity in TPJ drives behavior. TPJ is the ventral region in our network model and has critically been related to detecting unattended behaviorally relevant events such as invalidly cued targets and mismatches between predicted and observed inputs (Corbetta, Patel, & Shulman, 2008; Mengotti, Käsbauer, Fink, & Vossel, 2020).
- All three regions drive behavior. This model was included to test whether a mix of all brain regions most plausibly describes behavior, despite the additional complexity.

Bayesian model comparison was conducted with random effects (RFX) in the VBA‐toolbox (*VBA_groupBMCbtwConds*) and fixed effects (FFX) to select the most likely output region (IPS, TPJ, FEF, or all).

After selecting the most plausible output region, we inverted a classical DCM model by setting the prior mean and variance of the parameter set ψ to 0, essentially disabling the additional parameters necessary to fit behavioral dynamics. This included both parameters in the observation and evolution function. Moreover, we inverted models that included predictions from the behavioral Rescorla–Wagner model.

The following models were compared:

- bDCM (with output region selected as described above)
- DCM: The bDCM model above, but with the prior mean and variance of the evolution (i.e., $A_{r}$) and observation parameters set to 0, so that they are not considered in the model inversion. This model was chosen to compare the single modality model of fMRI and test whether bDCM merits its additional complexity.
- bDCM + Rescorla–Wagner (bDCM + RW): In this model, we added an additional input stream ($u_{3}$), including the RT predictions derived by the Rescorla–Wagner model. The input‐stream, however, was not included in the DCM part of the bDCM (i.e., A, B, C, and D) but was directly gated to the output function via the $C_{r}$ matrix. This model was included to assess if bDCM's predictions have additional value compared with the Rescorla–Wagner model's predictions.
- bDCM infused with the Rescorla–Wagner model (bDCM × RW): In this model, we replaced the input coding for invalid trials ($u_{2}$) with the prediction of the cue invalidity ($v_{\left(t-1\right)}$) of the Rescorla–Wagner model—note that this input was also centered. This tested whether the cognitive processes modeled by the Rescorla–Wagner model provide information over and above bDCM.

#### 2.6.3 Model evaluation of the Rescorla–Wagner model, classical DCM, and bDCM

In addition to the Bayesian model selection, we compared the models based on their outputs, applying classical goodness of fit statistics. The *R* ^2^‐score,

$$SS_{\mathrm{tot}}=\sum_{t}^{n}{\left(y_{t}-\bar{y}\right)}^{2}$$

$$SS_{\mathrm{res}}=\sum_{t}^{n}{\left(y_{t}-{\hat{y}}_{t}\right)}^{2}\;$$

$$R^{2}=1-\left(\frac{SS_{\mathrm{res}}}{SS_{\mathrm{tot}}}\right)$$

where $y_{t}$ describes the datapoint at t, with n time points in total, was calculated. The average of *y* is defined as $\bar{y}$, and $\hat{y}$ are predicted values. Similarly, we also calculated the mean absolute error (MAE)

$$\mathrm{MAE}=\frac{1}{n}\;\sum_{t}^{n}\left|y_{t}-{\hat{y}}_{t}\right|$$

Here, we estimated for each subject whether the fit statistics were different from random for each of the model outputs by permutation testing. The predicted values $\hat{y}$ were shuffled 10,000 times (without replacement), and the two statistics were recalculated. The permutation *p*‐value for the models is then reported as the proportion of fits greater than the model's *R* ^2^‐score (smaller in case of MAE) plus one divided by the number of permutations plus one (Ojala & Garriga, 2010). At the group level, we report the proportion of significant models based on a permutation *p*‐value <.05.

To compare the model performance on reaction times, we used (Bayesian‐) paired *t*‐tests to test for differences between the fit statistics [*R* ^2^‐score and mean absolute error (MAE)], separately for the two runs. In Section S7 in Supporting Information, we also present the results of a Leave‐One‐Trial‐Out cross‐validation procedure on the behavioral data of both the Rescorla–Wagner and the bDCM models to compare the generalization between the two models. A modified version of the *VBA_press.m* function was used to calculate predicted residual error sum of squares (PRESS) statistics.

We then investigated how well bDCM and the Rescorla–Wagner model simulate the underlying reaction time distributions. This was achieved by calculating a two‐sample Kolmogorov–Smirnov test between the model‐derived reaction times of the Rescorla–Wagner model or bDCM and the measured reaction times. Furthermore, paired *t*‐tests were used on the distance between the distributions (as determined by the KS‐test) to test which simulation followed the measured data more closely (i.e., had a smaller distance at the group level).

Finally, we were also interested in whether there are “spillover effects” from the previous trial in the reaction time data (i.e., how valid and invalid cues or errors in the previous trial affected the reaction time in the current trial). We used a linear mixed‐effects model with participant as a random factor as in other analyses. We also tested whether the stimulus onset asynchrony (SOA, 600 or 800 ms) influenced reaction time data using the same models. Notably, we performed this analysis on both the measured reaction times and the simulated reaction times of the Rescorla–Wagner and bDCM to investigate how the two modeling approaches represent trial history effects.

We applied a mixed‐effects linear model for each error term to compare differences in performance to the BOLD data fit between classical DCM and bDCM. The mixed‐effects model followed the following formula, where “Score” either depicts the mean absolute error or the *R* ^2^‐score:

Score~Model+Region+Run+Model*Region+Model*Run

and Model has the two factors “DCM” and “bDCM,” “Run” describes either the horizontal or vertical run, and “Region” indicates the “Score” for either VOI. Each model also contained a random intercept for each participant.

### 2.7 Lesion analysis

We also applied lesion analysis to the bDCM model, as described in Rigoux and Daunizeau (2015). Here, the afferent connections toward a single brain region were reduced to 0 to simulate the absence of this region (i.e., to create an artificial lesion). The simulated data from such a lesioned model can be used to better understand behavioral changes after damage to certain brain regions.

As mentioned above, much of our knowledge about the brain regions involved in the Posner task is based on lesions studies. Hence, based on the literature, we would assume that lesions to the right (and potentially also left) TPJ lead to an increase in reaction times toward invalidly cued targets in the contralesional hemifield, whereas the remaining conditions should be relatively unaffected (Beume et al., 2017; Corbetta & Shulman, 2011). For lesions to the left and right IPS, we expected extinction‐like patterns with prolonged reaction times for contralesional stimuli. However, just as TPJ lesions, focal IPS lesions can also lead to reorienting deficits in invalid trials (Gillebert et al., 2011; Posner et al., 1984). Lesion evidence for FEF is scarce, but TMS studies have shown that interference with right FEF leads to a general decrease in performance in both hemifields, whereas damage to left FEF only affects targets in the contralesional hemifield (Duecker & Sack, 2015; Hung, Driver, & Walsh, 2011).

For effects along the vertical meridian, the evidence is unfortunately very scarce. While some studies have found extinction of stimuli, particularly for the lower‐left visual field after right parietal lesions (Müri, Cazzoli, Nyffeler, & Pflugshaupt, 2009), others found neglect of the upper‐left visual field after lesions to the right temporal lobe (Morris, Mańkowska, & Heilman, 2020). Classical altitudinal or vertical neglect has more often been attributed to bilateral lesions (Rapcsak, Cimino, & Heilman, 1988; Shelton, Bowers, & Heilman, 1990).

Since numerous models were numerically unstable in the lesion analysis, we cleaned the simulated data by removing datasets on a per lesion basis where the variance after the 10th trial was close to 0 (i.e., the simulated reaction times flatlined at the maximum/minimum of the sigmoid function) and which returned nonvalues.

## 3 RESULTS

### 3.1 Behavior

**FIGURE 3:**
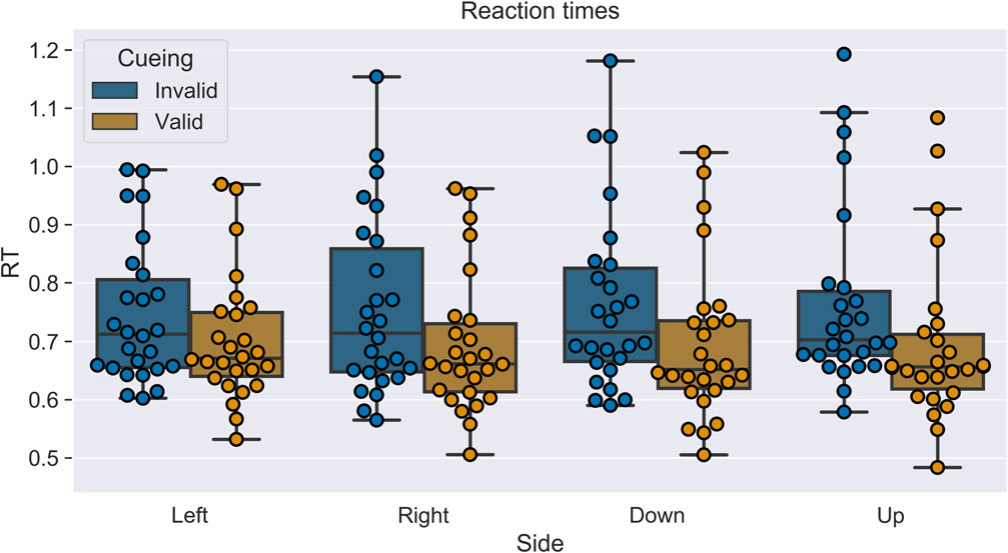
Box‐ and swarm plots of mean‐reaction time data for each participant in the eight conditions. The boxes indicate the interquartile range (IQR), the line in the middle the median reaction time, whiskers are extended to include the lower and upper quartiles plus three times the IQR. Loose points indicate outliers. The ANOVA's results are readily visible, as there are longer reaction times in invalid trials but no apparent effects between the different target‐positions

To test for reaction time effects of cueing (valid or invalid) and target‐location (left, right, down, up), as well as their interactions, we applied a 2 × 4 repeated measures ANOVA (see also Figure 3). There was no significant effect of target‐side [*F*(1.965, 49.125) = 0.1, *p* = .902, $\eta_{p}^{2}=0.004$, *ε* = 0.655]. However, a significant main effect of cueing [*F*(1, 25) = 26.647, *p* < .001, $\eta_{p}^{2}=0.516$, *ε* = 1.0] and a weak significant interaction between target‐side and cueing [*F*(2.88, 72) = 2.866, *p* = .045, $\eta_{p}^{2}=0.103$, *ε* = 0.96) were observed. All reported *p*‐values were Greenhouse–Geisser‐corrected to account for a lack of sphericity. An additional analysis confirmed that the experimental design's stimulus‐onset‐asynchrony did not impact our experimental data (Section S3 in Supporting Information).

### 3.2 FMRI GLM

**FIGURE 4:**
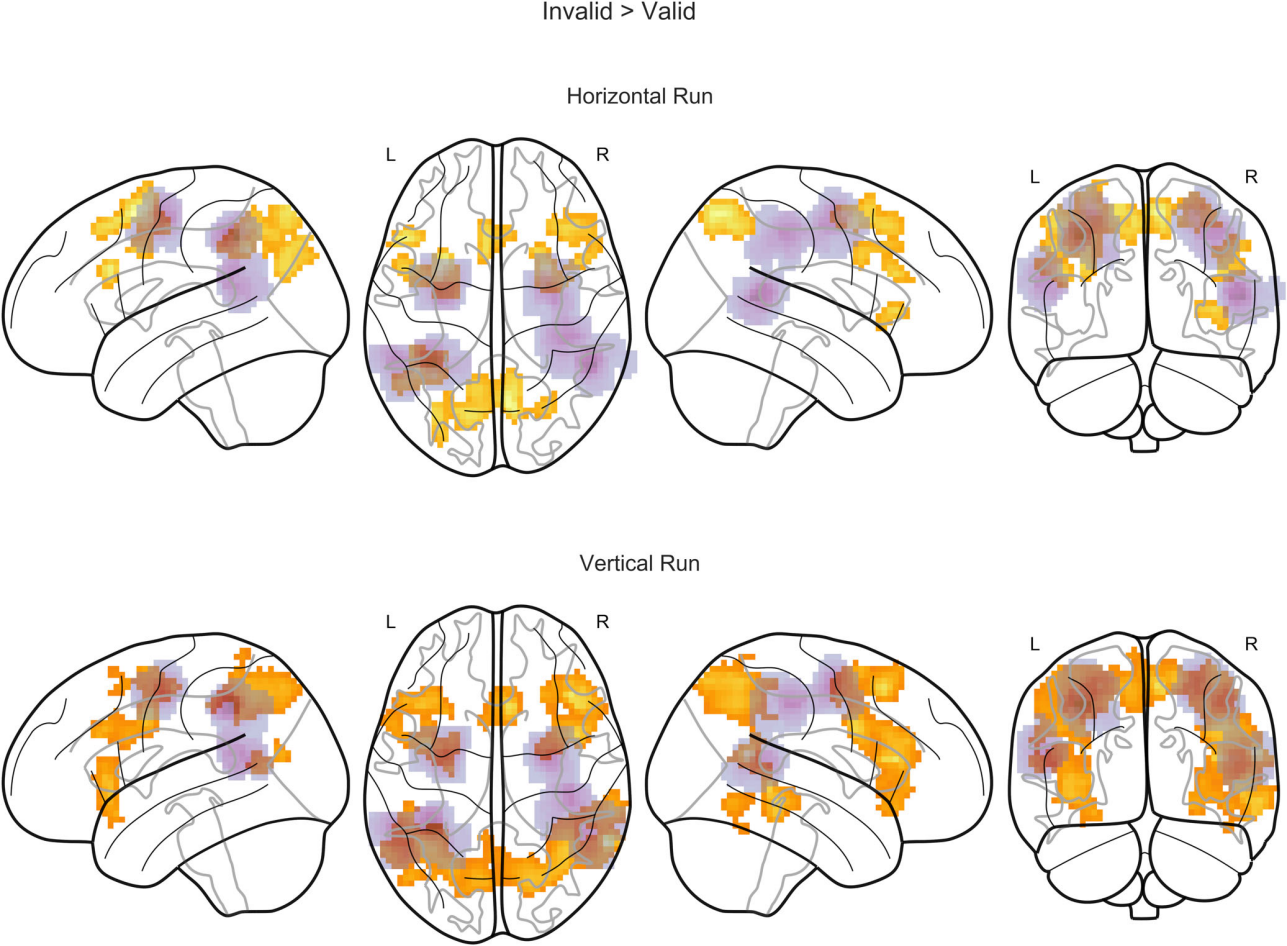
Nonparametric T‐maps contrasting invalid > valid trials for the two runs (*p* < .05 FWEc). The purple overlay indicates the regions where the 9 mm VOIs for the bDCM analysis were extracted (sum of the participants' masks)

The contrasts of invalid versus valid trials isolating reorienting‐related activity for the two runs are reported in Figure 4 (group t‐maps are provided on neurovault: https://identifiers.org/neurovault.collection:6895, [Gorgolewski et al., 2015], the corresponding tables reporting global and local maxima for the different clusters are in Section S2 in Supporting Information). We performed a one‐sample permutation *t*‐test on the first‐level contrast images (invalid > valid), with a predefined cluster‐forming threshold of *p* < .001, the results are reported family‐wise error corrected at *p* < .05 (cluster threshold horizontal *k* = 54 voxels, vertical *k* = 61 voxels). We found areas classically associated with the dorsal and ventral attention networks in both maps. For example, we observed significant activations in bilateral intraparietal sulci and frontal‐eye fields in both runs. Activations of the ventral attention networks were less robust. For the horizontal run, for example, the invalid versus valid contrast revealed an involvement of the middle frontal gyrus predominantly in the right hemisphere and no significant activation close to the seed regions for the temporoparietal junction at the given threshold. However, the temporoparietal junction was significantly activated in the vertical run.

### 3.3 Bayesian model selection

#### 3.3.1 Output regions of the bDCM

**FIGURE 5:**
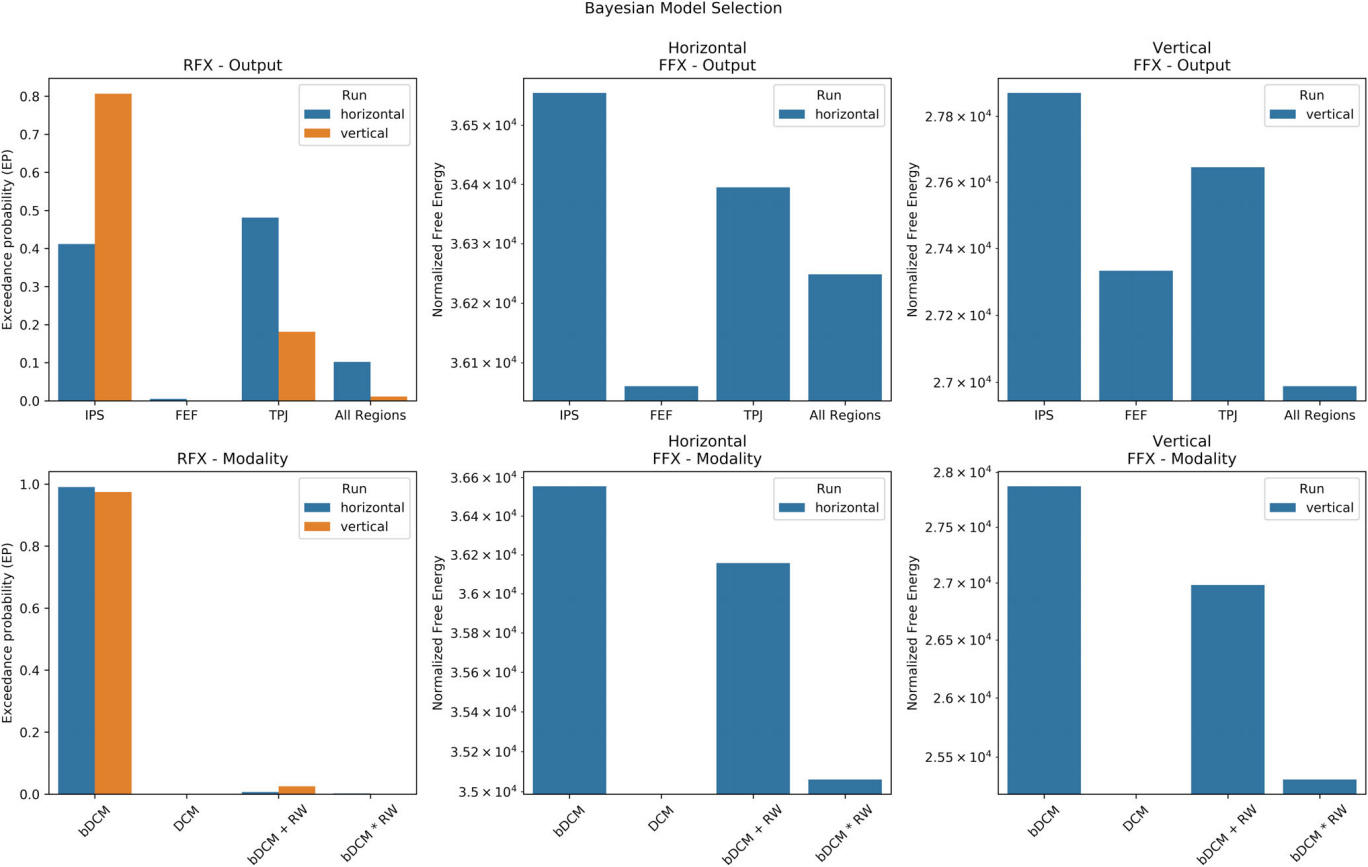
Bayesian model selection for the output regions (top row) and modality (bottom row). Due to the different scaling FFX model evidence, the horizontal and vertical runs are plotted separately. The models were normalized by subtracting the log likelihood of the Null‐Model. Please note that the *y*‐axis for the FFX analysis is on a logarithmic scale

As shown in the top row of Figure 5, RFX Bayesian model selection identified different winning models for the two runs. While in the vertical run, the IPS output model was superior to the models with other regions [exceedance probability (EP) = 81%], results for the horizontal run did not dissociate between the TPJ (EP = 48%) and the IPS model (EP = 41%). To select a single model structure for further analyses, we also conducted an FFX analysis (as implemented in SPM12) to investigate which model had the highest total free energy. This analysis revealed that evidence in both runs was around 100% for the IPS only model (free energy summed over participants for the horizontal run: IPS = −5,340.80, FEF = −5,834.72, TPJ = −5,500.46, all regions = −5,647.16; the vertical run: IPS = −21,946.42, FEF = −22,485.02, TPJ = −22,172.91, all regions = −22,829.19).

#### 3.3.2 Model selection for modalities

Testing for the different modalities (Figure 5, bottom row) revealed that the bDCM model (with IPS as output region) had stronger model evidence in both runs when compared with the competing models (horizontal, EP = 99%; vertical, EP = 97%). This indicates that bDCM provides a more plausible explanation of the data, even when adjusting for the additional complexity.

### 3.4 Model fit

#### 3.4.1 Reaction time data

**FIGURE 6:**
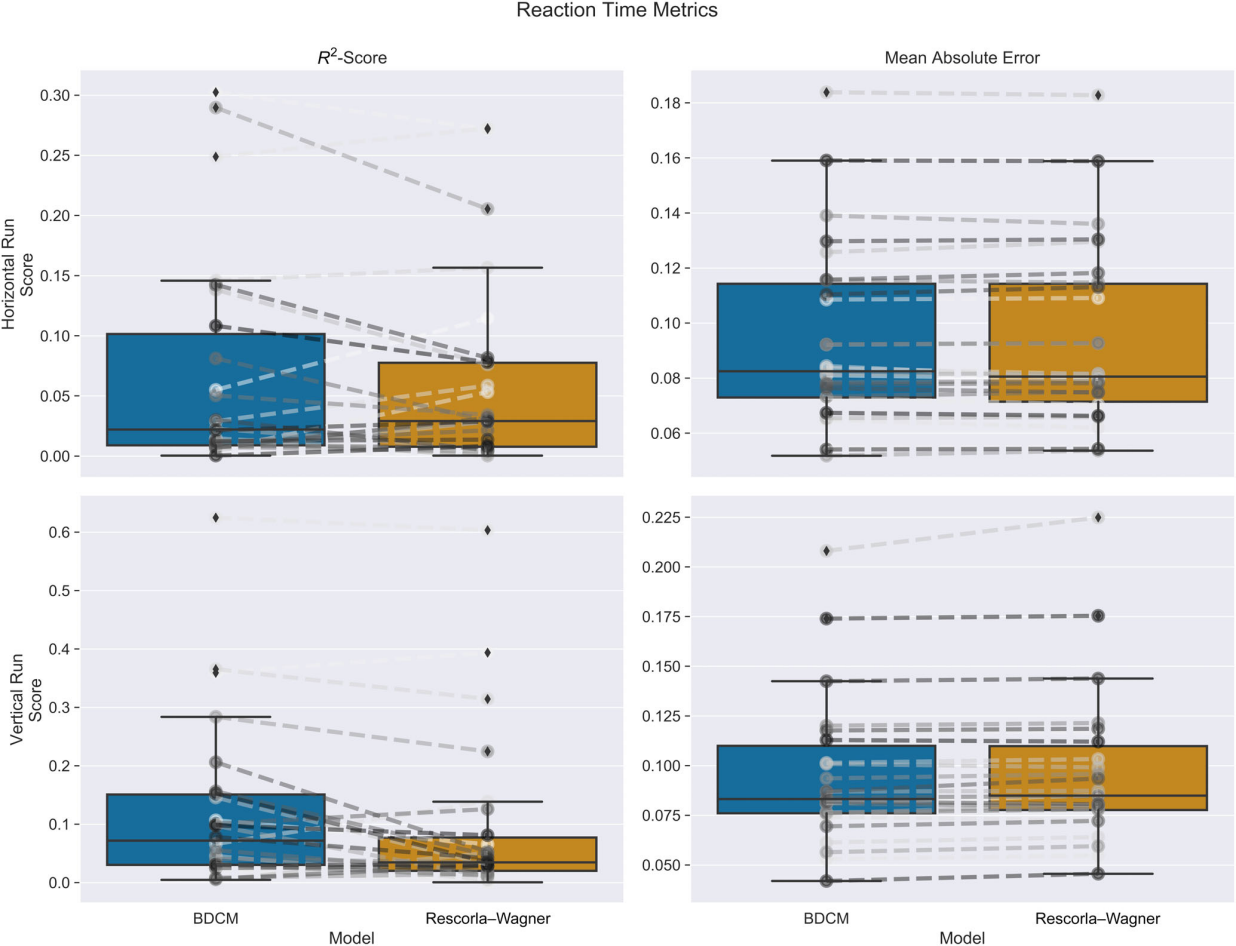
Boxplots comparing the different fit statistics across models in the horizontal run. Please note that for the *R* ^2^‐score a higher value is better, while the opposite is true for the mean absolute error. The dashed lines between the boxplots indicate individual participants

We calculated the mean absolute error (MAE) and the *R* ^2^ statistic for the Rescorla–Wagner and bDCM models and assessed their significance on a per‐subject level by calculating permutation tests. In the horizontal run, the bDCM (MAE, *M* = 0.094, *SD* = 0.033, percent sig. = 50.0%; *R* ^2^ score, *M* = 0.068, *SD* = 0.091, percent sig. = 57.7%) performed similarly, when compared with the Rescorla–Wagner model (MAE, *M* = 0.094, *SD* = 0.033, percent sig. = 50.0%; *R* ^2^ score, *M* = 0.062, *SD* = 0.08, percent sig. = 65.4%). The results of the vertical run yielded a very similar picture indicating slight differences between the bDCM (MAE, *M* = 0.095, *SD* = 0.037, percent sig. = 84.6%; *R* ^2^ score, *M* = 0.122, *SD* = 0.144, percent sig. = 88.5%) and the Rescorla–Wagner model (MAE, *M* = 0.096, *SD* = 0.038, percent sig. = 73.1%; *R* ^2^ score, *M* = 0.094, *SD* = 0.142, percent sig. = 84.6%; see Figure 6). The paired *t*‐tests (Table 3) confirmed this pattern and revealed a better fit for the vertical run, where bDCM had a lower error and greater fit than the Rescorla–Wagner model. In contrast to the comparison between regular fit‐statistics, the results of a Leave‐One‐Trial‐Out Cross‐validation (Section S7 in Supporting Information) showed that the Rescorla–Wagner model generalizes slightly better than bDCM.

**TABLE 3:** Paired *t*‐tests between the fit statistics of bDCM and Rescorla–Wagner models

Run	Error	*T* (*df* 25)	*p*‐value	95% CI	Cohen‐*d*	BF10
Horizontal	MAE	0.496	.624	[−0 0]	0.006	0.232
Horizontal	*R* ^2^	0.82	.42	[−0.01 0.02]	0.064	0.281
Vertical	MAE	−2.496	.019	[−0 −0]	0.049	2.705
Vertical	*R* ^2^	2.664	.013	[0.01 0.05]	0.197	3.713

*Note*: The differences between the fit statistics favor the bDCM model.

**FIGURE 7:**
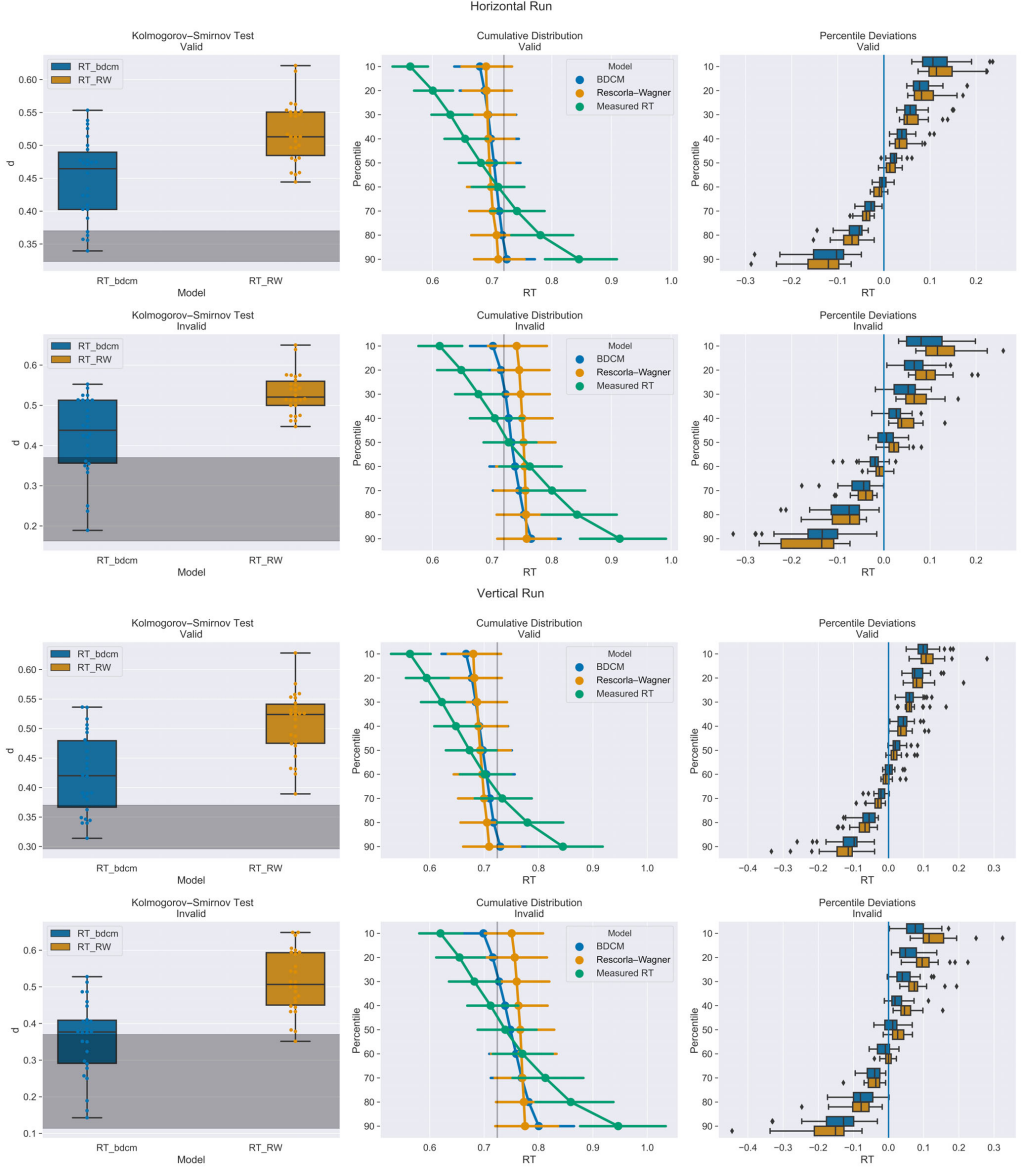
Left column, Kolmogorov–Smirnov distance between the simulated responses between the measured reaction times and bDCM or the Rescorla–Wagner model. The gray shading indicates nonsignificant differences. BDCM has, in general, a lower distance to the original distribution, and in many cases, the tests were nonsignificant. Both models also appeared to be better in predicting the invalid reaction time distribution. Middle column: Cumulative reaction time distribution represented by the deciles of each model. The measured reaction times (green) have a more extensive spread than the reaction times from bDCM (blue) and the Rescorla–Wagner model (orange). The gray vertical bar indicates the mean RT across deciles, cueing condition, and models. Right column: The paired difference between the deciles of the Rescorla–Wagner (orange) model and bDCM (blue). Differences are especially large in more extreme deciles

Furthermore, we evaluated how well the reaction time distributions of the two models' simulations matched the real reaction time distribution (Figure 7). We calculated the distance between the distributions of measured and simulated reaction times for each run, participant, and cueing‐condition using the Kolmogorov–Smirnov test. We then performed paired *t*‐tests in order to ascertain which simulation better matches the original distribution. In all cases, the bDCM simulation provided a better match (horizontal run, valid cueing, *t*(25) = −5.011, *p* < .001, Cohen's *d* = 1.316, BF_10_ = 667.1; horizontal run, invalid cueing, *t*(25) = −5.198, *p* < .001, Cohen's *d* = 1.357, BF_10_ = 1,033.7; vertical run, valid cueing, *t*(25) = −5.145, *p* < .001, Cohen's *d* = 1.391, BF_10_ = 913.9; vertical run, invalid cueing, *t*(25) = −6.059, *p* < .001, Cohen's *d* = 1.634, BF_10_ = 7,704.4). Based on visual inspection of the differences in reaction time distributions, deviations were especially pronounced at the extreme ends of the distribution.

In a final step, we also analyzed to which extent trial history effects (i.e., effects of cueing and errors in preceding trials) are modeled by the Rescorla–Wagner model and bDCM. The detailed results of this analysis can be found in Section S3 in Supporting Information. As expected, SOA did not significantly affect the measured reaction time data in any of the analyses. More interestingly, valid cues in the previous trial had a facilitating effect on reaction times of 11 ms (95% CI [−18 ms, −4 ms]), which was also present in the reaction times derived from the Rescorla–Wagner model (−7 ms, 95% CI [−9 ms, −5 ms]) and the bDCM (−5 ms, 95% CI [−8 ms, −3 ms]), indicating that both models captured trial‐by‐trial dependencies. We also found an effect of previous error‐trials in the measured data, impeding performance in the following trials (14 ms, 95% CI [0 ms, 27 ms]). This effect was not present in the reaction times of the Rescorla–Wagner model and the bDCM since error trials were not explicitly modeled.

#### 3.4.2 BOLD data

We also investigated whether DCM and bDCM were comparable in their fit to the measured BOLD data. For this, we calculated the fit statistics (mean absolute error and *R* ^2^‐score) for the two modeling approaches and the two runs. Since these yield fit statistics for each brain region, we calculated two linear mixed‐effects models (MLM) with participant as a random factor to test for a main effect or interaction effects of model and fit statistic, as well as the main effect of run and interactions between model and run. The results of the mixed‐effects models are summarized in Table 4. Importantly, we did not find a significant main effect of model. However, there were significant main effects of brain region. These effects did not interact with the choice of model, indicating that the two models performed similarly. A similar conclusion can be drawn when looking at the different runs. Again, there were no significant main effects or interactions of the factor model.

**TABLE 4:** Results of the MLM analysis for BOLD fit statistics

	*R* ^2^ score				Mean absolute error			
	Coef.	*SE*	*Z*	*p* > |*z*|	Coef.	*SE*	*z*	*p* > |*z*|
Intercept	.18	0.012	15.416	0	.316	0.014	22.755	0
DCM	.001	0.013	0.059	.953	0	0.016	−0.03	.976
FEF right	−.046	0.012	−3.83	0	−.032	0.015	−2.14	.032
IPS left	−.052	0.012	−4.345	0	−.054	0.015	−3.539	0
IPS right	−.063	0.012	−5.265	0	−.072	0.015	−4.748	0
TPJ left	−.072	0.012	−5.957	0	.029	0.015	1.895	.058
TPJ right	−.076	0.012	−6.347	0	−.008	0.015	−0.505	.613
Vertical run	.002	0.007	0.251	.802	.03	0.009	3.429	.001
DCM * FEF right	0	0.017	0.005	.996	0	0.021	0.013	.99
DCM * IPS left	.003	0.017	0.176	.86	0	0.021	−0.003	.997
DCM * IPS right	.004	0.017	0.261	.794	0	0.021	−0.006	.996
DCM * TPJ left	−.003	0.017	−0.173	.863	.001	0.021	0.04	.968
DCM * TPJ right	−.006	0.017	−0.368	.713	.002	0.021	0.094	.925
Vertical run * DCM	.005	0.01	0.476	.634	0	0.012	−0.038	.97

*Note*: The table is split for mean absolute error and *R* ^2^ score.

### 3.5 Lesion analysis

**FIGURE 8:**
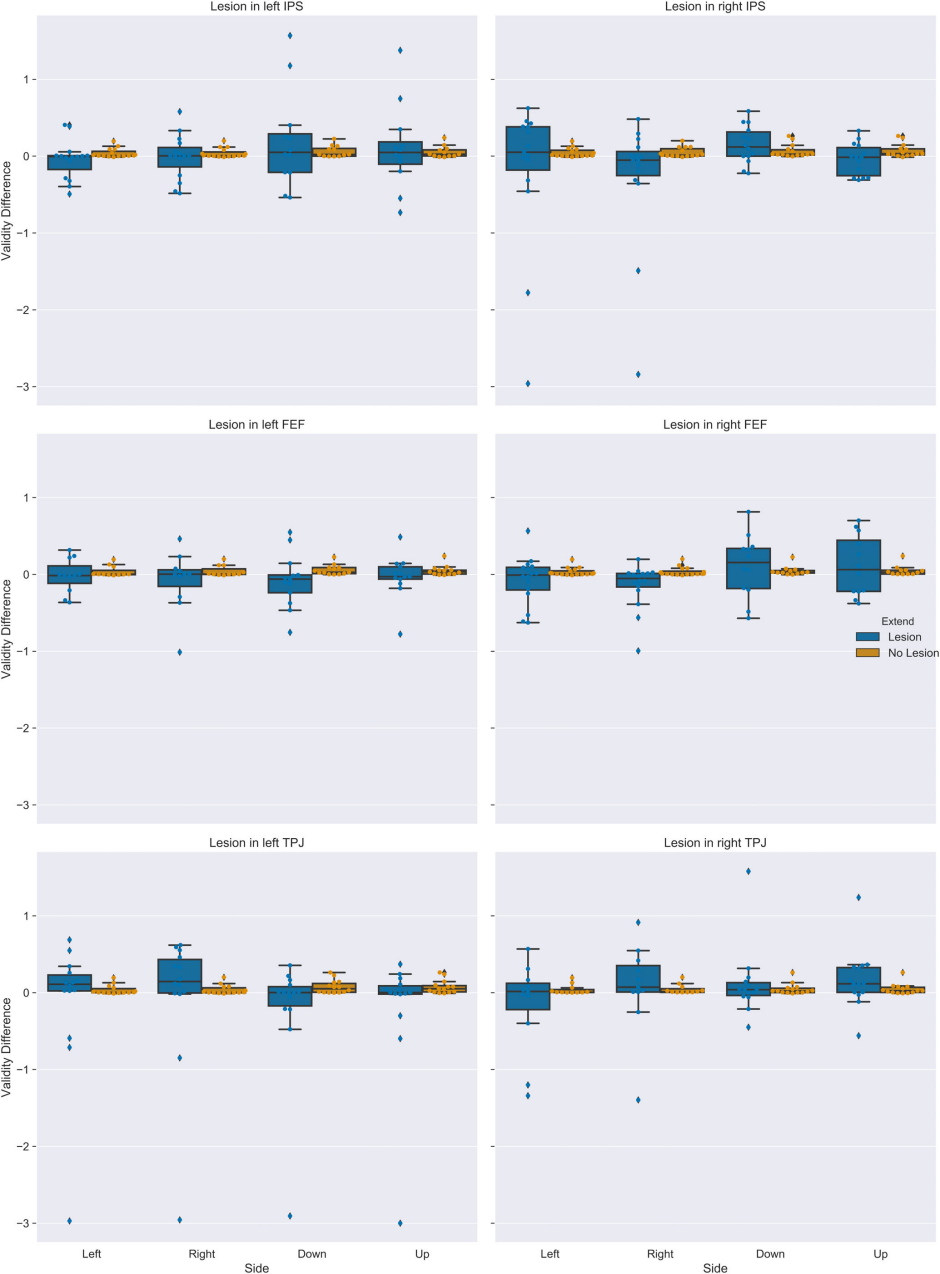
Validity effect for simulated reaction times for each target location after simulated lesions to the indicated brain region. Boxplots indicate the median of the data, the IQR, and the minimum and maximum values. Outliers exceed the 1.5 × IQR criterion

Figure 8 depicts the results of the lesion analysis for each possible target location (i.e., both runs are presented) and each induced artificial lesion. The effect of lesions on the validity effect seemed highly specific for the different network nodes with some lesions increasing and other lesion decreasing the validity effect.

From a computational anatomy perspective, lesioning the left TPJ yielded plausible effects, increasing the contralesional validity effect, similarly to what would be expected (Beume et al., 2017; Malherbe et al., 2018). A similar pattern was observed for the right IPS. However, simulations for this region were very noisy.

## 4 DISCUSSION

We applied bDCM (Rigoux & Daunizeau, 2015) to model neural responses and reaction times simultaneously in a spatial cueing task. We here demonstrated that bDCM could be applied to binary responses and continuous read‐outs (i.e., reaction times). After reproducing previously published cue validity effects at the behavioral and neural level, we selected the most likely output region for the behavioral response in the bDCM model and modeled behavioral and functional imaging data in three different ways.

First, in terms of RFX Bayesian model comparison, different output regions between the two runs were preferred. Relatively clear evidence in the vertical run pointed to a preference for the IPS, a central hub region of the dorsal attention network. However, in the horizontal run, there was no clear evidence for either IPS or TPJ (a key region of the ventral attention network). Still, the FFX model comparison favored the IPS model in both runs. A possible reason for this discrepancy might be that the target location influences neural states in IPS in the vertical run less compared with the horizontal run due to the lateralization of the visual field. Thus, for some participants, neural states in TPJ might provide a more apparent separation between valid and invalid trials in the horizontal run. However, the more substantial evidence for the dorsal hub‐region (i.e., IPS) in the FFX analysis also speaks for a better representation of reaction times and BOLD data by neural responses in IPS, compared with the other regions in our comparison (i.e., FEF and TPJ).

Furthermore, bDCM, as a novel approach, was compared with both classical DCM and the behavioral Rescorla–Wagner model. As the three models serve different purposes and rely on different data, we analyzed the models' outputs and fit statistics. We also accounted for differences in model complexity by using Bayesian model selection to compare bDCM against DCM and two bDCM models, which utilized different aspects of the Rescorla–Wagner model.

Although the original article on bDCM suggested that incorporating behavior leads to an advantage in representing the BOLD response of bDCM over classical DCM, we did not find significant differences between both modeling approaches. The benefit of including behavioral measures might only be prevalent when BOLD recordings are noisier than behavioral recordings (Rigoux & Daunizeau, 2015).

Furthermore, we compared simulated reaction times of bDCM and our implementation of the Rescorla–Wagner model (Vossel, Mathys, et al., 2014). BDCM had a slightly better fit to the reaction time data (reflected in higher *R* ^2^‐score and lower error) and represented the distribution of reaction times a bit more closely in both valid and invalid trials (reflected in significantly lower distances, which were calculated by the Kolmogorov–Smirnov test). Both the Rescorla–Wagner model and bDCM did not model the extreme ends of the reaction time distributions well. However, bDCM deviated less from the measured data. Interestingly, bDCM can similarly to the Rescorla‐Wagner model capture trial‐by‐trial effects present in the measured data, without explicitly modelling them.

This comparison was not performed to favor one model over the other. Instead, it was conducted to evaluate bDCM against the performance of a highly specialized, validated, and less complex model in a cueing task. Despite the superior fit of bDCM, the Rescorla–Wagner model performed exceptionally well, given the small number of parameters. Hence, if we penalized for model complexity, the Rescorla–Wagner model would probably be identified as the preferred model for reaction times. In an additional analysis (Section S7 in Supporting Information), we provided evidence for this notion: the Rescorla–Wagner has a slightly (but significantly) better generalization performance (as assessed by the PRESS statistic). BDCM also incorporates the dynamics of the BOLD response and operates on a timescale of seconds rather than trials. Thus, having only four parameters more than the classical DCM (69 parameters) seems to be an adequate increase in complexity. We further validated this notion by incorporating the Rescorla–Wagner model's results into competing bDCM models and performing a Bayesian model comparison. This revealed that the initial bDCM was superior to models in which hidden states or predicted reaction times were guided through the bDCM.

BDCM also provided a more detailed representation of reaction time distributions. This property might be helpful to uncover relevant aspects for assessing cognitive functions, as previously demonstrated for other modeling approaches. For example, parameters of drift‐diffusion models of reaction times (Smith & Ratcliff, 2009) were found to be related to general intelligence (van Ravenzwaaij, Brown, & Wagenmakers, 2011) and working memory (Schmiedek, Oberauer, Wilhelm, Süß, & Wittmann, 2007). Furthermore, reaction time distributions have been used to categorize healthy participants and patients suffering from psychiatric disorders (Kaiser et al., 2008; Karalunas, Geurts, Konrad, Bender, & Nigg, 2014; Vinogradov, Poole, Willis‐Shore, Ober, & Shenaut, 1998). The Rescorla–Wagner model could also be used for such differentiations, especially in the domain of belief‐updating (Mengotti et al., 2017). By modeling a single cognitive process, however, the Rescorla–Wagner model is very dependent on the presence and size of a participant's validity effects (see Analysis in Section S4 in Supporting Information, showing that the correlation between model fit and cue‐validity are higher for Rescorla–Wagner than bDCM).

BDCM, on the other hand, simulated smoother reaction time distributions (larger number of nonsignificant *p*‐values in KS‐test), providing a different representation of the underlying processes. Although bDCM may reflect a portion of variance in the reaction time data that is not task‐related, this variance could reflect the processes of decision making in a more complex brain‐dynamics‐dependent matter. BDCM is a *model of brain dynamics* that can, in principle, be applied to any task, while the Rescorla–Wagner model represents a specialized *model of a cognitive process*.

In another analysis, we demonstrate that reaction times simulated by both the Rescorla–Wagner model and bDCM are significantly influenced by the cueing condition of the previous trial. This influence is also found in the measured reaction time data (Section S3 in Supporting Information). This is important, as it would have been possible to use different behavioral models for our comparisons, such as drift‐diffusion models (Ratcliff & McKoon, 2008). However, while the Posner task could be modeled in terms of evidence accumulation for the respective side where the stimulus might occur, drift‐diffusion models do not necessarily capture essential aspects of the task (such as trial history and learning effects). Indeed, multiple studies have shown that endogenous cueing tasks (like Posner's cueing task) are not affected by manipulation of information gathering (for example, stimulus properties) (Barbot, Xue, & Carrasco, 2020; Carrasco, 2011) and that behavioral effects are mostly driven by expectation effects (Eckstein, Shimozaki, & Abbey, 2002). Thus, the Rescorla–Wagner model seems more suited to model the temporal dependencies between trials.

As bDCM can be applied to model different behavioral read‐outs in various tasks, it can enhance our understanding of how DCM's connectivity parameters relate to behavior. So far, this link could only be established using indirect methods, such as correlations between DCM parameters and behavioral measures across participants. For example, DCM's task connectivity parameters have been related to symptoms of depression and schizophrenia (Desseilles et al., 2011; Schlösser et al., 2008; Wu et al., 2014), and have been correlated with behavioral measures before and after interventions using noninvasive neurostimulation (Grefkes et al., 2010). Although the investigation of such associations does not allow causal interpretations, bDCM enables more firm conclusions on how brain dynamics in selected brain regions impact behavior.

Furthermore, brain and behavioral dynamics both regularize bDCM, so that the model parameters encode the most reliable set of information from both sources (Rigoux & Daunizeau, 2015). This procedure could yield more robust and stable connectivity estimates and encode more specific information.

Since bDCM is a generative model, it can also be used to simulate how alterations to the underlying brain network might change behavior (Rigoux & Daunizeau, 2015). This allows simulating the behavioral effects of neuromodulatory interventions and the generation of new hypotheses and experiments. The guidance and information of computational models will eventually lead to a better understanding of the neural mechanisms underlying behavioral outcomes (Kriegeskorte & Douglas, 2018; Turner et al., 2017).

Unfortunately, applying artificial lesions to the network model in our study revealed technical problems of this approach. More specifically, the estimated models lacked numerical stability, limiting our results' meaning and interpretability. Even though some of the resulting patterns were consistent with the literature, for example, an increase of the contralesional validity effect after a lesion to left TPJ (Beume et al., 2017; Malherbe et al., 2018), other simulations were highly variable. Hence, the relatively novel bDCM approach's potential problems, such as over‐fitting and nongeneralizability, need to be considered in future studies.

## 5 CONCLUSION

BDCM was applied and extended to model reaction time data of a larger sample of participants. Our findings provided evidence for a considerable additional value of the method compared with a purely behavioral model and classical DCM and identified practical use issues. Data suggest that bDCM is a promising tool to enhance our understanding of how brain dynamics generate specific behavioral patterns.

## CONFLICT OF INTERESTS

The authors declare no competing financial interests.

## Supporting information


**Appendix S1** Supporting Information.

## Data Availability

The data that support the findings of this study are available upon reasonable request from the corresponding author. The data are not publicly available due to privacy or ethical restrictions.
