# Supplementary material for: Simultaneous modeling of reaction times and brain dynamics in a spatial cueing task
Source: Hum Brain Mapp. 2021 Dec 24;43(6):1850–67. doi: 10.1002/hbm.25758 (PMC8933333; doi:10.1002/hbm.25758)
Supplement: Supplementary file 1 — Appendix S1 Supporting Information. [file HBM-43-1850-s001.docx]

# S1 Details for fMRIprep

The preprocessing of functional and anatomical data was performed using FMRIPREP version 1.1.1 (Esteban et al., 2018, 2019, RRID:SCR_016216), a Nipype (RRID:SCR_002502, Gorgolewski et al., 2011, 2017) based tool, run as a docker-image. Each T1-weighted volume (T1w) was corrected for intensity non-uniformity using N4BiasFieldCorrection v2.1.0 (Tustison et al., 2010) and skull-stripped using antsBrainExtraction.sh v2.1.0 (using the OASIS template). Spatial normalization to the ICBM 152 Nonlinear Asymmetrical template version 2009c (Fonov, Evans, McKinstry, Almli, & Collins, 2009, RRID:SCR_008796) was performed through nonlinear registration with the antsRegistration tool of ANTs v2.1.0 (Avants, Epstein, Grossman, & Gee, 2008, RRID:SCR_004757), using brain-extracted versions of both T1w volume and template. Brain tissue segmentation of cerebrospinal fluid (CSF), white matter (WM), and gray matter (GM) was performed on the brain-extracted T1w using fast (Zhang, Brady, & Smith, 2001, FSL v5.0.9, RRID:SCR_002823).

Functional data were slice-time corrected using 3dTshift from AFNI v16.2.07 (Cox, 1996, RRID:SCR_005927) and motion-corrected using mcflirt (FSL v5.0.9, Jenkinson, Bannister, Brady, & Smith, 2002). "Fieldmap-less" distortion correction was performed by co-registering the functional image to the same-subject T1w image with intensity inverted (Wang et al., 2017) constrained with an average fieldmap template (Treiber et al., 2016), implemented with antsRegistration (ANTs). This was followed by co-registration to the corresponding T1w using boundary-based registration (Greve & Fischl, 2009) with 9 degrees of freedom, using flirt (FSL). Motion correcting transformations, field distortion correcting warp, BOLD-to-T1w transformation, and T1w-to-template (MNI) warp were concatenated and applied in a single step using antsApplyTransforms (ANTs v2.1.0) using Lanczos interpolation.

Frame-wise displacement (Power et al., 2014) was calculated for each functional run using the implementation of Nipype.

Many internal operations of FMRIPREP use Nilearn (Abraham et al., 2014, RRID:SCR_001362), principally within the BOLD-processing workflow. For more details of the pipeline, see http://fmriprep.readthedocs.io/en/1.1.1/workflows.html.

# S2 fMRI – GLM analysis coordinates

Table 1 and Table 2 for the peak coordinates shown in Figure 4 of the main manuscript. The contrast invalid > valid is shown for each run of the fMRI experiment separately. We used the Harvard-Oxford atlas to label the regions and reported the region with maximum probability.

| Table S1: Peak statistics and clusters sizes for the horizontal T-map. Labels were automatically extracted from the Harvard-Oxford atlas, reporting the region with the highest probability from the coordinates. | | | | | | |
| --- | --- | --- | --- | --- | --- | --- |
| **Horizontal Run** | | | | | | |
| **Cluster ID** | **X** | **Y** | **Z** | **Peak Stat**  **(T)** | **Cluster Size (mm3)** | **Label** |
| 1 | -27.25 | 8.62 | 57.3 | 5.57 | 8250 | Superior Frontal Gyrus |
| 1a | -46 | 5.5 | 37.5 | 4.43 |  | Inferior Frontal Gyrus, pars opercularis |
| 1b | -24.12 | 2.38 | 73.8 | 3.85 |  | Insular Cortex |
| 1c | -39.75 | -3.88 | 47.4 | 3.68 |  | Inferior Frontal Gyrus, pars opercularis |
| 2 | 29 | 2.38 | 54 | 5.56 | 4350 | Superior Frontal Gyrus |
| 2a | 22.75 | 14.88 | 54 | 4.51 |  | Insular Cortex |
| 3 | 7.12 | -69.5 | 57.3 | 5.4 | 16886 | Cingulate Gyrus, posterior division |
| 3a | -14.75 | -75.75 | 57.3 | 4.77 |  | Angular Gyrus |
| 3b | -33.5 | -75.75 | 34.2 | 4.57 |  | Angular Gyrus |
| 3c | -8.5 | -60.12 | 47.4 | 4.41 |  | Cingulate Gyrus, posterior division |
| 4 | -49.12 | 21.12 | 27.6 | 5.11 | 2030 | Inferior Frontal Gyrus, pars triangularis |
| 5 | -33.5 | -53.88 | 37.5 | 5.08 | 6219 | Postcentral Gyrus |
| 5a | -42.88 | -50.75 | 47.4 | 5.07 |  | Supramarginal Gyrus, posterior division |
| 5b | -30.38 | -44.5 | 37.5 | 4.07 |  | Supramarginal Gyrus, anterior division |
| 6 | -5.38 | 14.88 | 54 | 4.73 | 3319 | Subcallosal Cortex |
| 6a | 7.12 | 24.25 | 54 | 4.11 |  | Insular Cortex |
| 7 | 38.38 | 27.38 | 4.5 | 4.55 | 1869 | Occipital Fusiform Gyrus |
| 8 | 41.5 | 27.38 | 24.3 | 4.5 | 3706 | Superior Frontal Gyrus |
| 8a | 44.62 | 8.62 | 34.2 | 4.26 |  | Inferior Frontal Gyrus, pars opercularis |
| 8b | 50.88 | 24.25 | 40.8 | 3.93 |  | Superior Frontal Gyrus |

| Table S2: Peak statistics and clusters sizes for the vertical T-map. Labels were automatically extracted from the Harvard-Oxford atlas, reporting the region with the highest probability from the coordinates. | | | | | | |
| --- | --- | --- | --- | --- | --- | --- |
| **Vertical Run** | | | | | | |
| **Cluster ID** | **X** | **Y** | **Z** | **Peak Stat**  **(T)** | **Cluster Size (mm3)** | **Label** |
| 1 | 60.25 | -53.88 | 17.7 | 7.18 | 8475 | Supramarginal Gyrus, posterior division |
| 1a | 54 | -41.38 | -2.1 | 5.62 |  | Middle Temporal Gyrus, posterior division |
| 1b | 63.38 | -38.25 | -8.7 | 5.5 |  | Middle Temporal Gyrus, posterior division |
| 2 | 41.5 | 27.38 | 14.4 | 6.29 | 23718 | Middle Frontal Gyrus |
| 2a | 44.62 | 5.5 | 30.9 | 5.81 |  | Inferior Frontal Gyrus, pars opercularis |
| 2b | 41.5 | 33.62 | 24.3 | 5.01 |  | Superior Frontal Gyrus |
| 2c | 29 | -0.75 | 54 | 5 |  | Superior Frontal Gyrus |
| 3 | 7.12 | 21.12 | 54 | 6.09 | 5091 | Insular Cortex |
| 3a | -5.38 | 11.75 | 57.3 | 4.02 |  | Insular Cortex |
| 4 | -36.62 | -0.75 | 40.8 | 6 | 12310 | Inferior Frontal Gyrus, pars opercularis |
| 4a | -24.12 | -13.25 | 54 | 5.02 |  | Inferior Frontal Gyrus, pars opercularis |
| 4b | -33.5 | -3.88 | 57.3 | 4.84 |  | Superior Frontal Gyrus |
| 4c | -49.12 | 24.25 | 30.9 | 4.75 |  | Superior Frontal Gyrus |
| 5 | 38.38 | -57 | 57.3 | 5.39 | 35707 | Angular Gyrus |
| 5a | -36.62 | -53.88 | 47.4 | 5.23 |  | Postcentral Gyrus |
| 5b | 19.62 | -72.62 | 57.3 | 5.19 |  | Angular Gyrus |
| 5c | 35.25 | -60.12 | 44.1 | 5.19 |  | Angular Gyrus |
| 6 | -39.75 | 21.12 | -5.4 | 5.18 | 4930 | Frontal Pole |
| 6a | -33.5 | 24.25 | 1.2 | 4.89 |  | Frontal Pole |
| 6b | -27.25 | 18 | -15.3 | 4.57 |  | Cuneal Cortex |
| 6c | -42.88 | 24.25 | 11.1 | 4.29 |  | Middle Frontal Gyrus |
| 7 | -46 | -72.62 | 17.7 | 4.59 | 2255 | Angular Gyrus |
| 7a | -33.5 | -72.62 | 17.7 | 4.3 |  | Angular Gyrus |
| 7b | -58.5 | -60.12 | 14.4 | 4.21 |  | Angular Gyrus |
| 7c | -46 | -63.25 | 11.1 | 3.63 |  | Lateral Occipital Cortex, superior division |
| 8 | 57.12 | -63.25 | -8.7 | 4.46 | 2062 | Lateral Occipital Cortex, superior division |
| 8a | 44.62 | -63.25 | -12 | 3.93 |  | Lateral Occipital Cortex, superior division |

# S3 Analysis of trial-by-trial dependencies

In a further analysis, we investigated how the previous trial influences reaction times in each trial. Different from previous analyses, we primarily modeled error trials in this analysis. The following tables are Linear Mixed-Effects Models results with random intercepts for each participant, conducted using the statsmodels api in Python.

| Table S3: Results of mixed-effects analysis for the measured reaction time data. | | | | | | |
| --- | --- | --- | --- | --- | --- | --- |
| **Factor** | **Coef.** | **Std.Err.** | **z** | **P>\|z\|** | **[0.025** | **0.975]** |
| **Cueing – Invalid** | 0.766 | 0.024 | 31.446 | 0 | 0.718 | 0.813 |
| **Cueing – Valid** | 0.705 | 0.024 | 29.149 | 0 | 0.657 | 0.752 |
| **Cueing_Before – Valid** | -0.011 | 0.004 | -3.094 | 0.002 | -0.018 | -0.004 |
| **Error_Before** | 0.014 | 0.007 | 1.995 | 0.046 | 0 | 0.027 |
| **SOA (long)** | -0.001 | 0.003 | -0.378 | 0.705 | -0.007 | 0.004 |
| **Run – vertical** | 0 | 0.003 | 0.172 | 0.864 | -0.005 | 0.006 |
| **Group Var** | 0.015 | 0.03 |  |  |  |  |

| Table S4: Results of mixed-effects analysis for simulated reaction time data of the bDCM. | | | | | | |
| --- | --- | --- | --- | --- | --- | --- |
| **Factor** | **Coef.** | **Std.Err.** | **z** | **P>\|z\|** | **[0.025** | **0.975]** |
| **Cueing – Invalid** | 0.744 | 0.024 | 31.13 | 0 | 0.697 | 0.791 |
| **Cueing – Valid** | 0.704 | 0.024 | 29.488 | 0 | 0.657 | 0.751 |
| **Cueing_Before – Valid** | -0.005 | 0.001 | -4.34 | 0 | -0.008 | -0.003 |
| **Error_Before** | 0.001 | 0.002 | 0.273 | 0.785 | -0.004 | 0.005 |
| **SOA (long)** | 0.001 | 0.001 | 1.105 | 0.269 | -0.001 | 0.003 |
| **Run – vertical** | 0 | 0.001 | 0.361 | 0.718 | -0.002 | 0.002 |
| **Group Var** | 0.015 | 0.084 |  |  |  |  |

| Table S5: Results of mixed-effects analysis for simulated reaction time data of the Rescorla-Wagner model. | | | | | | |
| --- | --- | --- | --- | --- | --- | --- |
| **Factor** | **Coef.** | **Std.Err.** | **z** | **P>\|z\|** | **[0.025** | **0.975]** |
| **Cueing – Invalid** | 0.762 | 0.024 | 31.921 | 0 | 0.715 | 0.809 |
| **Cueing – Valid** | 0.702 | 0.024 | 29.408 | 0 | 0.655 | 0.748 |
| **Cueing_Before – Valid** | -0.007 | 0.001 | -7.02 | 0 | -0.009 | -0.005 |
| **Error_Before** | 0.003 | 0.002 | 1.553 | 0.121 | -0.001 | 0.007 |
| **SOA (long)** | 0 | 0.001 | -0.099 | 0.921 | -0.002 | 0.002 |
| **Run – vertical** | 0 | 0.001 | 0.561 | 0.575 | -0.001 | 0.002 |
| **Group Var** | 0.015 | 0.1 |  |  |  |  |

As seen in tables S3-S5, bDCM and the Rescorla-Wagner model capture the influence of the previous trial on the current trial, showing that expectation is a crucial factor in Posner’s spatial cueing paradigm.

# S4 Correlation between fit statistic and validity differences

To test whether the size of the validity effect influenced the resulting fit statistic, we correlated (using Pearson’s correlations) each permutation of error (R^2^, mean absolute error), model (Rescorla-Wagner), and run with the validity difference (mean RT invalid – mean RT valid). The R^2^-score was highly correlated with the validity difference, where the effect appeared to be stronger for the Rescorla-Wagner model than for the bDCM. There was, however, no correlation with the mean absolute error (MAE).

| Table S6: Pearson’s Correlations between the validity difference and the different fit statistics. | | | | | | |
| --- | --- | --- | --- | --- | --- | --- |
| **Model** | **Score** | **Run** | **r** | **p** | **Adj. R2** | **BF_10_** |
| bDCM | MAE | Horizontal | 0.22 | 0.281 | -0.034 | 0.423 |
|  |  | Vertical | 0.105 | 0.61 | -0.075 | 0.275 |
|  | R^2^ | Horizontal | 0.794 | 0 | 0.599 | 1.58E+04 |
|  |  | Vertical | 0.806 | 0 | 0.619 | 2.79E+04 |
| Rescorla-Wagner | MAE | Horizontal | 0.215 | 0.29 | -0.036 | 0.414 |
|  |  | Vertical | 0.108 | 0.598 | -0.074 | 0.278 |
|  | R^2^ | Horizontal | 0.829 | 0 | 0.659 | 9.61E+04 |
|  |  | Vertical | 0.858 | 0 | 0.713 | 6.34E+05 |


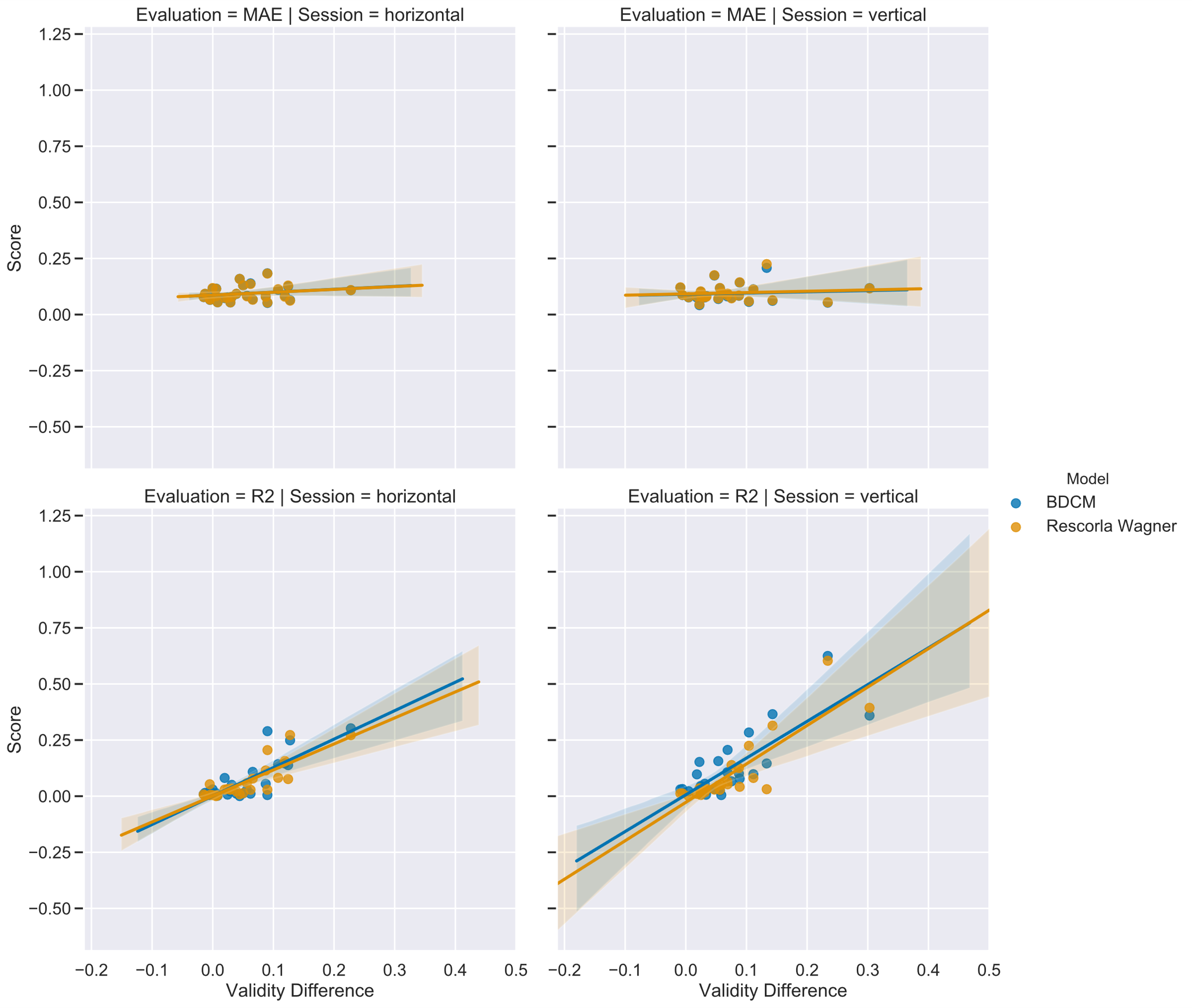


Figure S1: Pearson correlation between the validity difference (x-axis) and the fit statistics (u-axis) for the two models, the different metrics (rows), and the two runs (columns).

# S5 Connectivity matrices

Figures S3-S6 display the average connectivity strength of the (behavioral) dynamic causal modeling analysis. Connectivity parameters were averaged across participants. Inhibitory connections (A matrix diagonal) were exponentiated before averaging.


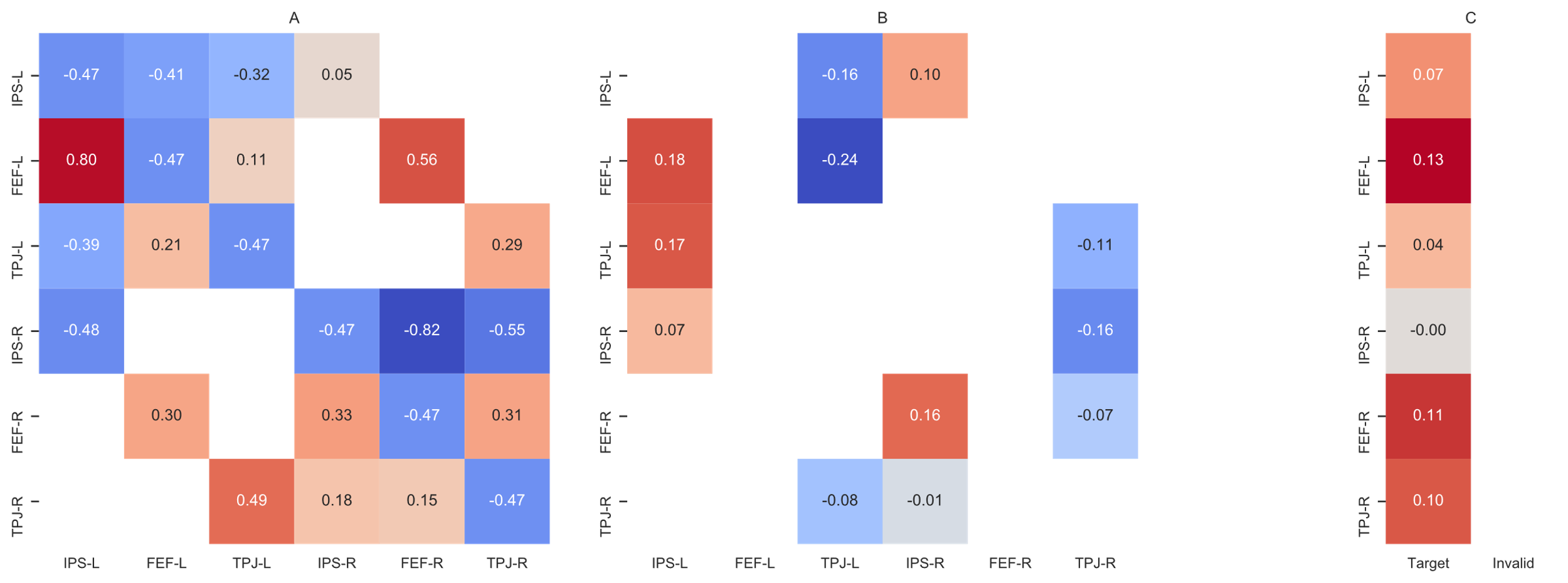


Figure S4: The average strength of the DCM connectivity parameters of the vertical run.


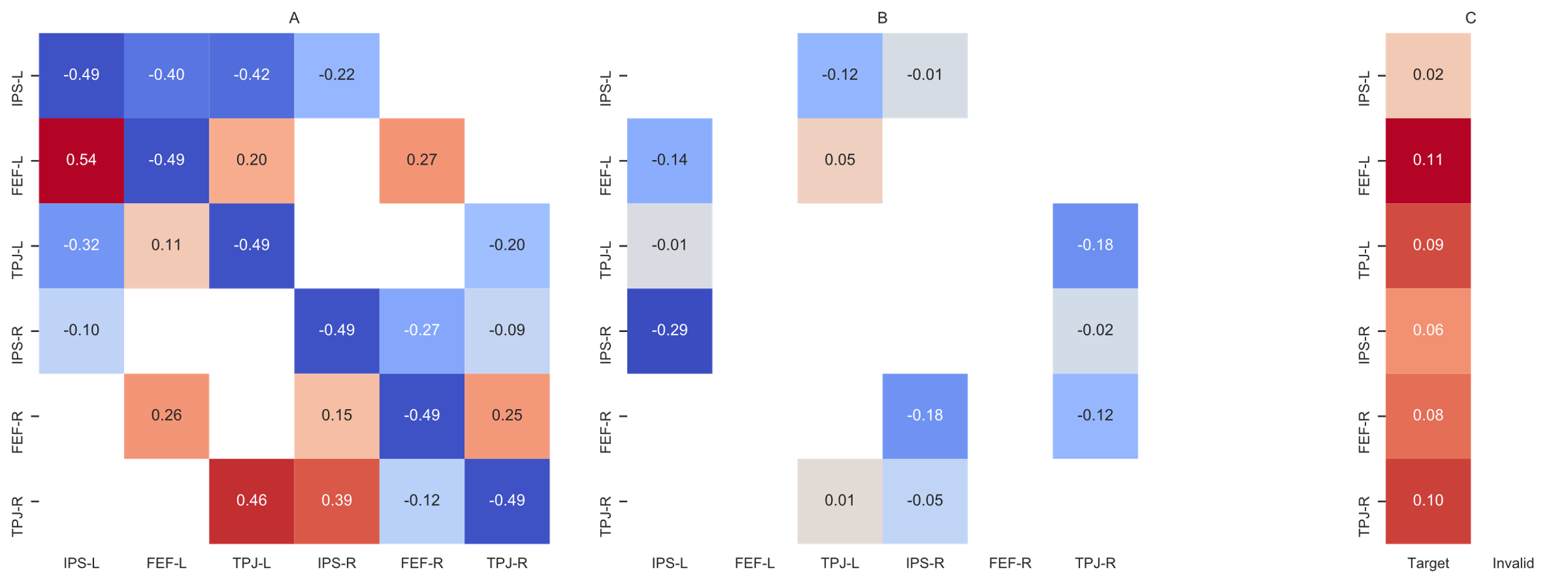


Figure S3: The average strength of the DCM connectivity parameters of the horizontal run.


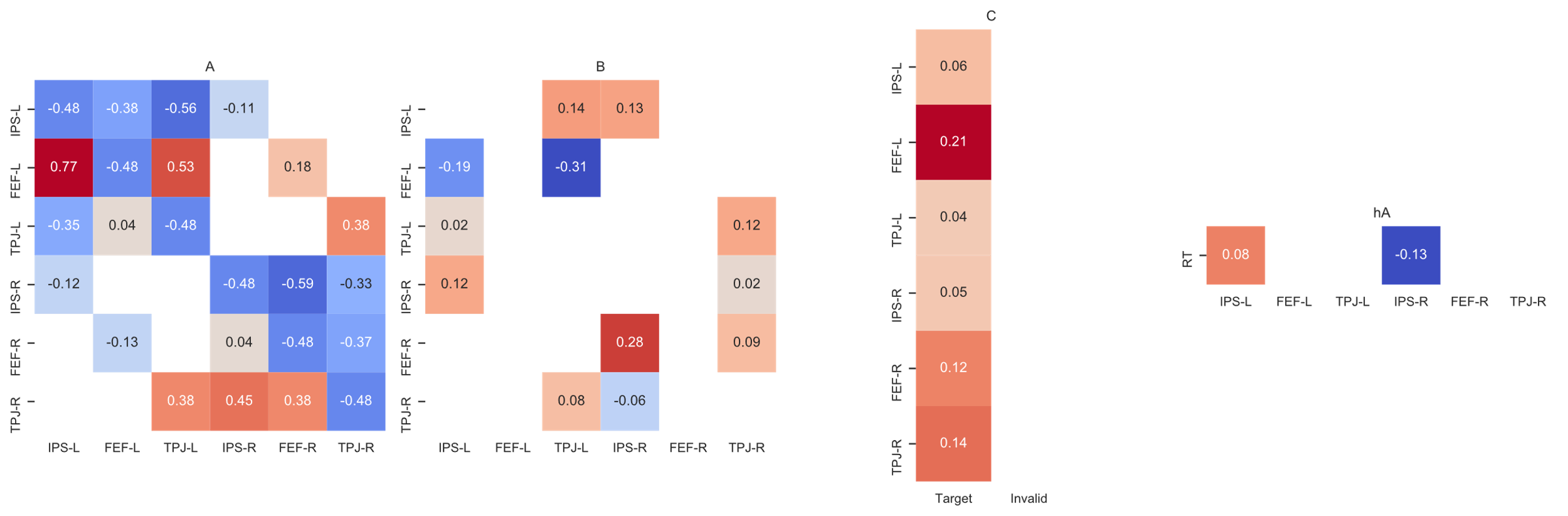


Figure S6: The average strength of the bDCM connectivity parameters of the vertical run.


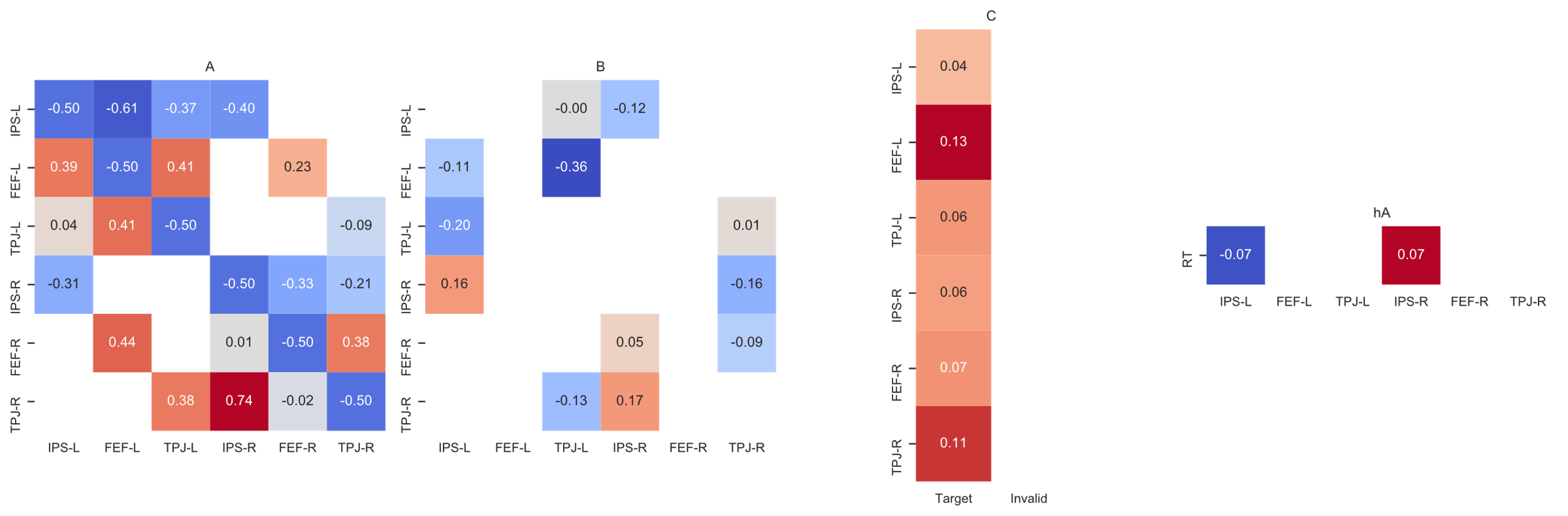


Figure S5: The average strength of the bDCM connectivity parameters of the horizontal run.

# S6 Parameter Estimates for the Rescorla-Wagner Model

Please note that the parameter estimates for $\boldsymbol{\zeta}_{\boldsymbol{2}}$ are negative, as the parametrization of our model predicts invalidly cued trials rather than validly cued trials. The correct expectation of an invalid cue thus leads to a faster reaction time.

| Table S7: Mean and SD over participants for the posterior estimates of the parameters included in the Rescorla-Wagner model. | | | | | |
| --- | --- | --- | --- | --- | --- |
| **Horizontal Run** | | | **Vertical Run** | | |
|  | Mean | SD |  | Mean | SD |
| $\alpha$ | 0.492 | 0.055 | $\alpha$ | 0.46 | 0.099 |
| $\zeta_{v}$ | 0.755 | 0.134 | $\zeta_{v}$ | 0.77 | 0.151 |
| $\zeta_{i}$ | 0.718 | 0.129 | $\zeta_{i}$ | 0.717 | 0.127 |
| $\zeta_{2}$ | -0.026 | 0.04 | $\zeta_{2}$ | -0.03 | 0.07 |

# S7 Leave-One-Out Cross-Validation

We also conducted leave-one-trial-out cross-validation for the Rescorla-Wagner and bDCM model. For this, we slightly adapted the VBA_press.m function in the VBA-toolbox to perform cross-validation over the behavioral data only. Our measure of interest was the predicted residual error sum of squares (PRESS) statistic, which is given by the formula below:

$$\mathrm{PRESS} = \sum_{i}^{n} \left( y_{i} - \hat{y}_{i,-i} \right)^{2}$$

Here $\hat{y}_{i,-i}$ describes the prediction of the model, that was fitted without the value i.


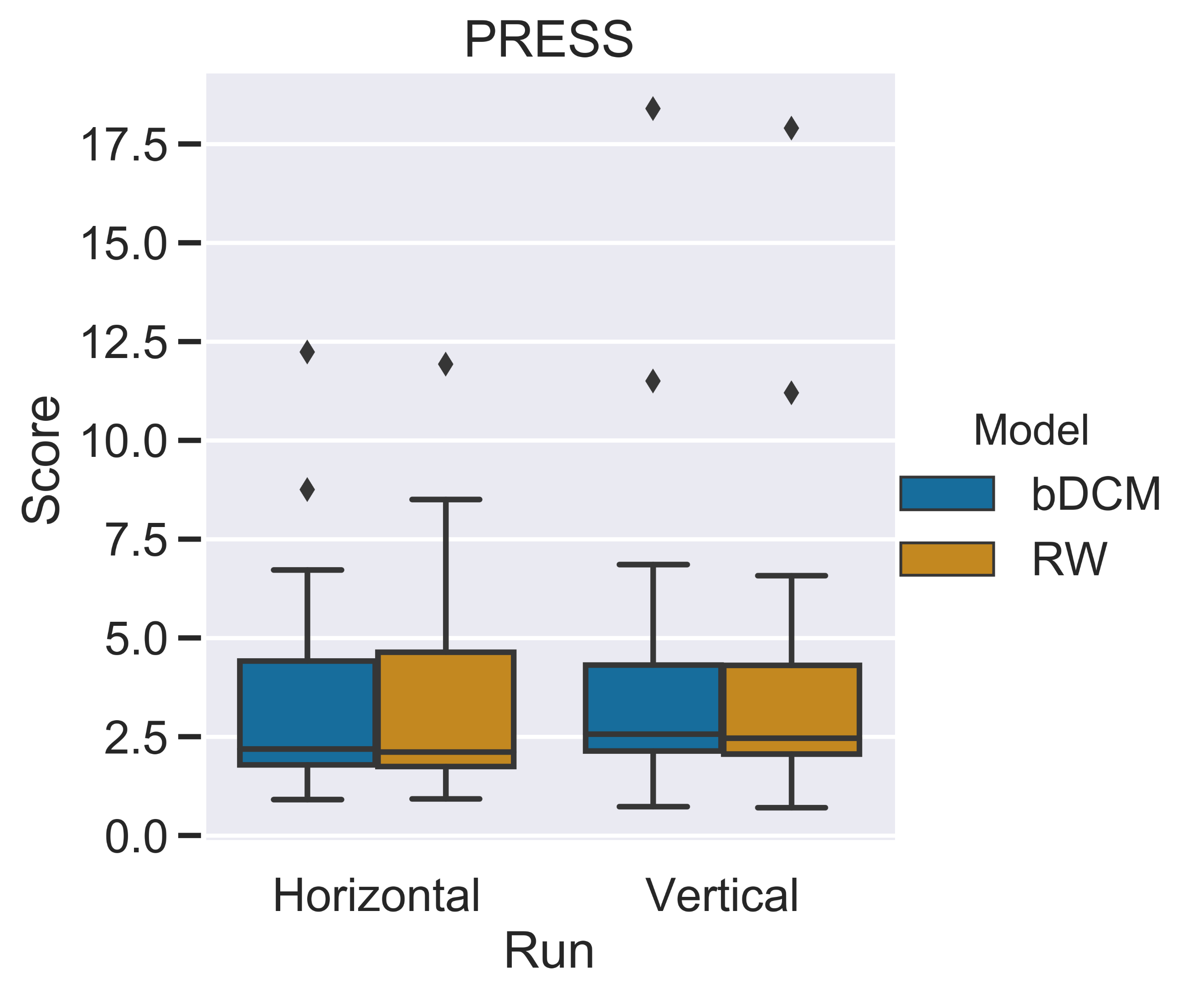


Figure S7: Comparison of PRESS statistics between the Rescorla-Wagner model and bDCM.

Fig. S7 and tables S8 and S9 indicate that the Rescorla-Wagner model has a slightly better generalization performance in terms of the PRESS statistics than bDCM. Although bDCM additionally incorporates the BOLD signal for each trial, it does not appear to have a significant regularization effect. Conversely, the Rescorla-Wagner model relies on only a few parameters, so individual missing values do not appear to have a significant effect. While systematic, the differences in performance between bDCM and Rescorla-Wagner are relatively small (Cohen’s d for horizontal = 0.03 and vertical 0.04). Still, it appears to be the case that bDCM is over-parameterized compared to the Rescorla-Wagner model.

| Table S8: PRESS statistic’s mean and SD for each run and model. | | | |
| --- | --- | --- | --- |
|  |  | **Mean** | **SD** |
| **Run** | **Model** |  |  |
| Horizontal | RW | 3.377 | 2.632 |
|  | bDCM | 3.473 | 2.714 |
| Vertical | RW | 3.712 | 3.615 |
|  | bDCM | 3.863 | 3.718 |

| Table S9: Paired t-test between the PRESS statistics of bDCM versus Rescorla-Wagner model. | | | | | | | |
| --- | --- | --- | --- | --- | --- | --- | --- |
|  | **T** | **DF** | **p** | **CI95%** | **Cohen’s-d** | **BF_10_** | **Test** |
| **bDCM > RW** | 2.126 | 25 | 0.044 | [0. 0.19] | 0.036 | 1.41 | Horizontal |
|  | 4.067 | 25 | 0.0004 | [0.07 0.23] | 0.0411 | 74.46 | Vertical |

# References

Abraham, A., Pedregosa, F., Eickenberg, M., Gervais, P., Mueller, A., Kossaifi, J., … Varoquaux, G. (2014). Machine learning for neuroimaging with scikit-learn. *Frontiers in Neuroinformatics*, *8*. doi: 10.3389/fninf.2014.00014

Avants, B., Epstein, C., Grossman, M., & Gee, J. (2008). Symmetric diffeomorphic image registration with cross-correlation: Evaluating automated labeling of elderly and neurodegenerative brain. *Medical Image Analysis*, *12*(1), 26–41. doi: 10.1016/j.media.2007.06.004

Cox, R. W. (1996). AFNI: Software for Analysis and Visualization of Functional Magnetic Resonance Neuroimages. *Computers and Biomedical Research*, *29*(3), 162–173. doi: 10.1006/cbmr.1996.0014

Esteban, O., Blair, R., Markiewicz, C. J., Berleant, S. L., Moodie, C., Ma, F., … Gorgolewski, K. J. (2018, July 31). *Poldracklab/Fmriprep: 1.1.3*. Zenodo. doi: 10.5281/zenodo.1323934

Esteban, O., Markiewicz, C. J., Blair, R. W., Moodie, C. A., Isik, A. I., Erramuzpe, A., … Gorgolewski, K. J. (2019). fMRIPrep: A robust preprocessing pipeline for functional MRI. *Nature Methods*, *16*(1), 111–116. doi: 10.1038/s41592-018-0235-4

Fonov, V., Evans, A., McKinstry, R., Almli, C., & Collins, D. (2009). Unbiased nonlinear average age-appropriate brain templates from birth to adulthood. *NeuroImage*, *47*, S102. doi: 10.1016/S1053-8119(09)70884-5

Gorgolewski, K. J., Burns, C. D., Madison, C., Clark, D., Halchenko, Y. O., Waskom, M. L., & Ghosh, S. S. (2011). Nipype: A Flexible, Lightweight and Extensible Neuroimaging Data Processing Framework in Python. *Frontiers in Neuroinformatics*, *5*. doi: 10.3389/fninf.2011.00013

Gorgolewski, K. J., Esteban, O., Ellis, D. G., Notter, M. P., Ziegler, E., Johnson, H., … Ghosh, S. (2017, May 21). *Nipype: A Flexible, Lightweight And Extensible Neuroimaging Data Processing Framework In Python. 0.13.1*. Zenodo. doi: 10.5281/zenodo.581704

Greve, D. N., & Fischl, B. (2009). Accurate and robust brain image alignment using boundary-based registration. *NeuroImage*, *48*(1), 63–72. doi: 10.1016/j.neuroimage.2009.06.060

Jenkinson, M., Bannister, P., Brady, M., & Smith, S. (2002). Improved Optimization for the Robust and Accurate Linear Registration and Motion Correction of Brain Images. *NeuroImage*, *17*(2), 825–841. doi: 10.1006/nimg.2002.1132

Power, J. D., Mitra, A., Laumann, T. O., Snyder, A. Z., Schlaggar, B. L., & Petersen, S. E. (2014). Methods to detect, characterize, and remove motion artifact in resting state fMRI. *NeuroImage*, *84*, 320–341. doi: 10.1016/j.neuroimage.2013.08.048

Treiber, J. M., White, N. S., Steed, T. C., Bartsch, H., Holland, D., Farid, N., … Chen, C. C. (2016). Characterization and Correction of Geometric Distortions in 814 Diffusion Weighted Images. *PLOS ONE*, *11*(3), e0152472. doi: 10.1371/journal.pone.0152472

Tustison, N. J., Avants, B. B., Cook, P. A., Yuanjie Zheng, Egan, A., Yushkevich, P. A., & Gee, J. C. (2010). N4ITK: Improved N3 Bias Correction. *IEEE Transactions on Medical Imaging*, *29*(6), 1310–1320. doi: 10.1109/TMI.2010.2046908

Wang, S., Peterson, D. J., Gatenby, J. C., Li, W., Grabowski, T. J., & Madhyastha, T. M. (2017). Evaluation of Field Map and Nonlinear Registration Methods for Correction of Susceptibility Artifacts in Diffusion MRI. *Frontiers in Neuroinformatics*, *11*. doi: 10.3389/fninf.2017.00017

Zhang, Y., Brady, M., & Smith, S. (2001). Segmentation of brain MR images through a hidden Markov random field model and the expectation-maximization algorithm. *IEEE Transactions on Medical Imaging*, *20*(1), 45–57. doi: 10.1109/42.906424
